# Engineering formation of multiple recombinant Eut protein nanocompartments in *E. coli*

**DOI:** 10.1038/srep24359

**Published:** 2016-04-11

**Authors:** Mark Held, Alexander Kolb, Sarah Perdue, Szu-Yi Hsu, Sarah E. Bloch, Maureen B. Quin, Claudia Schmidt-Dannert

**Affiliations:** 1 Department of Biochemistry, Molecular Biology and Biophysics, University of Minnesota, St. Paul, MN 55108, USA

## Abstract

Compartmentalization of designed metabolic pathways within protein based nanocompartments has the potential to increase reaction efficiency in multi-step biosynthetic reactions. We previously demonstrated proof-of-concept of this aim by targeting a functional enzyme to single cellular protein nanocompartments, which were formed upon recombinant expression of the *Salmonella enterica* LT2 ethanolamine utilization bacterial microcompartment shell proteins EutS or EutSMNLK in *Escherichia coli*. To optimize this system, increasing overall encapsulated enzyme reaction efficiency, factor(s) required for the production of more than one nanocompartment per cell must be identified. In this work we report that the cupin domain protein EutQ is required for assembly of more than one nanocompartment per cell. Overexpression of EutQ results in multiple nanocompartment assembly in our recombinant system. EutQ specifically interacts with the shell protein EutM *in vitro* via electrostatic interactions with the putative cytosolic face of EutM. These findings lead to the theory that EutQ could facilitate multiple nanocompartment biogenesis by serving as an assembly hub for shell proteins. This work offers insights into the biogenesis of Eut bacterial microcompartments, and also provides an improved platform for the production of protein based nanocompartments for targeted encapsulation of enzyme pathways.

One goal of synthetic biology is the expression of re-engineered metabolic pathways in heterologous hosts for the industrial scale production of valuable chemicals^1^,^2^. Significant advances have been made towards that goal owing to the increased availability of genome sequence data, as well as the development of affordable DNA synthesis technologies. However, one major challenge that still remains is the ability to achieve efficient metabolic flux in designed cellular reaction schemes^3^. It is becoming increasingly apparent that optimization of substrate and intermediate channeling between enzymes in the novel pathway is required. In nature, metabolic enzymes are often co-localized or compartmentalized within the cell to increase reaction efficiency^4^,^5^,^6^. Physical proximity between functionally related enzymes prevents loss of intermediates by diffusion while favoring substrate and product flux, thereby improving the overall reaction kinetics of the multi-step pathway^7^,^8^. Similar principles can also be applied in the optimization of productive engineered metabolic pathways, as well as *in vitro* biocatalysis^9^,^10^,^11^.

One method that bacteria use for spatial organization of metabolic pathways is the compartmentalization of enzymes in bacterial microcompartments (BMCs). These are large (100–200 nm in diameter) organelles that encapsulate thousands of copies of metabolic pathway enzymes within the lumen of a single-layered protein shell^12^,^13^,^14^,^15^,^16^,^17^. The outer shell of the BMC is composed of self-assembling protein hexamers and pentamers (belonging to the BMC domain family Pfam:00936 or EutN/CcmL family Pfam:03319)^18^,^19^,^20^,^21^,^22^,^23^,^24^,^25^,^26^,^27^,^28^,^29^,^30^,^31^ that interact as a mosaic of tiles to create a 2D lattice^32^, similar to viral capsids. Each tile has a central pore, which allows passage of substrates, products and co-factors between the interior and exterior of the BMC shell^33^,^34^,^35^, ensuring optimal functioning of encapsulated enzymes. The sequestration of enzymes within the interior of the shell, and in some cases initiation of outer shell formation^36^,^37^,^38^, is mediated by transient interactions between shell proteins and short targeting sequences located on the N- or C-termini of the encapsulated enzyme(s)^36^,^39^,^40^,^41^,^42^,^43^. Sequestration of metabolic pathways within BMCs serves to increase reaction efficiency and to prevent leakage of toxic pathway intermediates to the cytosol of the cell.

The major types of BMCs that have been characterized to date include the carboxysome which is involved in carbon fixation in chemoautotrophs and cyanobacteria^44^; and the 1,2-propanediol utilization (Pdu) and ethanolamine utilization (Eut) BMCs that enable growth of enteric bacteria like *Salmonella*, *Escherichia* and *Clostridium* in the intestine^13^,^14^. Bioinformatics studies have also identified newly characterized and putative gene clusters encoding BMC domain family proteins alongside enzymes involved in diverse metabolic pathways in a wide range of bacteria from different environments (e.g. the halophile *Haliangium ochraceum* produces particularly robust BMC shell proteins^45^,^46^), indicating that encapsulation of enzymes within BMCs may be a widespread evolutionary adaptive feature^20^,^22^,^45^,^47^,^48^,^49^,^50^,^51^,^52^,^53^,^54^. Recent efforts have focused on understanding the form and function of several different types of BMCs, and subsequently redesigning them as protein-based nanobioreactors for targeted enzyme encapsulation, with the goal of optimizing efficiency of novel metabolic pathways for biotechnological applications^41^,^46^,^55^,^56^,^57^,^58^,^59^,^60^,^61^,^62^,^63^,^64^,^65^,^66^.

Towards this aim, we recently developed a system for targeting cargo proteins to the interior of recombinant protein nanocompartments in the heterologous host *E. coli* C2566, using the Eut BMC shell proteins from *S. enterica* LT2. By expressing either the five shell proteins EutSMNLK, or the single shell protein EutS, under the control of our in-house pUCBB BioBrick™ modified *lac* promoter plasmid system which allows constitutive expression^67^, a single stationary nanocompartment was formed per *E. coli* cell. Cargo proteins, including the fluorescent protein EGFP and a functional enzyme β-galactosidase, could be targeted to, and encapsulated within, the nanocompartments by tagging with a short N-terminal peptide sequence (EutC^1–19^) that we identified on one of the native encapsulated Eut metabolic enzymes (ethanolamine ammonia lyase complex β-subunit EutC)^39^. This represented a straightforward model system for the recombinant production of tailor designed nanobioreactors for *in vivo* biocatalysis applications.

One aspect of our model system that could be optimized to provide a more versatile platform for efficient multi-step catalysis is the number of recombinant compartments formed per cell in a heterologous host. The native organism, *S. enterica* LT2, produces multiple^68^, and sometimes unconstrained Eut BMCs per cell when grown on ethanolamine to induce BMC formation^39^. This is in contrast to the single immobile nanocompartment that is formed upon recombinant expression of Eut shell proteins in the heterologous host *E. coli*^39^. We hypothesized that another gene encoded within the *eut* gene cluster of *S. enterica* LT2 must be involved in the biogenesis of multiple unconstrained BMCs, which was not present in our heterologous system. The goal of this work was therefore to identify which genetic elements of the *S. enterica* LT2 *eut* gene cluster are required for the biogenesis of multiple nanocompartments in our recombinant expression system, and to control the formation of these multiple compartments in a heterologous host such as *E. coli* which, unlike the human pathogen associated *S. enterica* (strain LT2 is highly attenuated for virulence^69^ and is therefore not recognized as a GRAS organism), is a widely used and accepted bacterial expression system for industrial biotechnology and biocatalysis. The results of this work offer valuable insights into the formation of Eut BMCs, as well as provide an optimized configuration for *in vivo* nanobioreactor assembly, which will serve as a basis for future biotechnological applications.

## Results

### Search for *eut* genes that may play a role in BMC biogenesis

Recombinant expression of the Eut shell proteins (EutS or EutSMNLK) in the heterologous host *E. coli* is not sufficient for multiple nanocompartment formation. To identify the genetic element(s) that may be required for the multiple^68^, unconstrained^39^ Eut BMC phenotype that has been observed in *S. enterica* LT2, we carried out bioinformatics searches. The genes encoding enzymes necessary for ethanolamine utilization by *S. enterica* LT2 are encoded on the >15-kb *eut* operon. This gene cluster is composed of 17 open reading frames encoding regulatory elements; the metabolic enzymes necessary for breakdown of ethanolamine to acetyl CoA, acetyl phosphate and ethanol; and the five BMC shell proteins EutSMNLK (reviewed in:^14^). Many of the genes in the *eut* cluster have been fully characterized by other groups, and have not been described as being required for multiple Eut BMC formation (see^14^ for details), ruling out their potential involvement in multiple BMC biogenesis. However, the potential role of the proteins encoded by three of the genes in the *eut* operon in biogenesis remain unclear, EutJ, EutP and EutQ.

EutJ (NP_461397.1), which is annotated as a putative chaperone-like protein similar to DnaK or Hsp70^70^, is not essential for growth of *S. enterica* LT2 on ethanolamine^71^. The function of EutJ is unknown. EutP (NP_461404.1) belongs to the EutP/PduV family and shares sequence homology with other members of the RAS-like GTPase superfamily (PF00071); which is a diverse family of small GTPases that are involved in signal transduction^72^. The N-terminal region of the EutP homolog PduV has been reported to target proteins to the outer surface of PduABJKNU BMCs heterologously expressed in *E. coli*^62^. EutP may play a similar role in targeting proteins to Eut BMCs, however, this remains to be verified. Finally, it has been suggested that EutQ (NP_461403.1) may play an unidentified role in enclosing the dehydrogenase EutE within *S. enterica* LT2 BMCs^73^. The crystal structure of an N-terminally truncated EutQ homolog, CD1925 from *Clostridium difficile* 630, reveals that the protein has a cupin barrel fold (members of this superfamily have diverse functions as dioxygenases, decarboxylases, isomerases or in small molecule binding^74^), but that it lacks the typical metal coordinating histidines at the putative ligand binding site^25^. Therefore from a structural point of view the function of EutQ remains uncertain. A study by Penrod and Roth^71^ concluded that deletion of *eut*P or *eut*Q from *S. enterica* LT2 does not affect overall growth on ethanolamine, but does result in an increased acetaldehyde release during growth on this carbon source. This would suggest that these two proteins may be involved in the formation of fully functional Eut compartments in *S. enterica* LT2. Very recently, while this manuscript was under review, *in vitro* studies revealed that EutP and EutQ display novel acetate kinase activity, despite both proteins displaying significant differences in size and sequence to other previously characterized acetate kinases. It appears from this study that the acetate kinase activity of EutQ is not essential for growth of *S. enterica* Typhimurium on ethanolamine^75^.

To explore whether any of the proteins EutJ, EutP, or EutQ could be involved in multiple nanocompartment biogenesis, we cloned and expressed them as part of our recombinant system in *E. coli*.

### Effect of recombinant expression of EutJ, EutP, and EutQ on nanocompartment formation in *E. coli*

To investigate the behavior of EutJ, EutP or EutQ in a heterologous host, we first cloned the genes individually into our in-house high copy number pUCBB BioBrick™ plasmid which allows for constitutive expression^67^, and soluble expression of the proteins in *E. coli* C2566 cells was confirmed (Supplementary Table 1, Supplementary Figure 1). Subsequently, we stacked EutJ, EutP or EutQ with the *S. enterica* LT2 shell proteins EutS or EutSMNLK on our pUCBB plasmid system (Supplementary Table 1). To observe the effect of EutJ, EutP and EutQ on EutS nanocompartment assembly *in vivo*, we coexpressed the constructs in *E. coli* C2566 (note that this lab strain contains a non-functional *eut* operon and is not capable of growth on ethanolamine (Supplementary Figure 2), and does not produce BMCs unless shell proteins are recombinantly overexpressed^39^), with our previously characterized targeting sequence-fluorescent reporter construct, EutC^1–19^-EGFP (on our in-house low copy number constitutive plasmid pACBB)^39^,^67^. This system allows for the straightforward and rapid confirmation of nanocompartment assembly by visualization of discrete fluorescent puncta by fluorescent microscopy.

**Figure 1:**
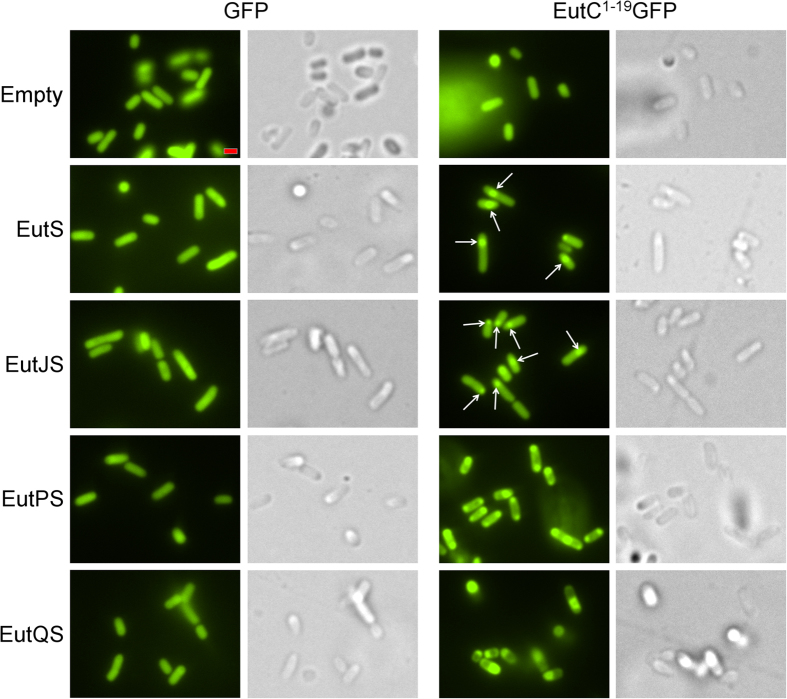
Effect of EutJ, EutP and EutQ expression on EutS nanocompartment assembly in *E. coli*. The left side of the panel displays cells co-expressing the shell protein EutS along with EutJ, P, or Q and an untagged EGFP reporter. Images on the right side show cells co-expressing EutS along with EutJ, P, or Q and the EutC^1–19^-EGFP fusion. Control cells (Empty: expressing an empty plasmid without *eut* genes) expressing cargo proteins alone are also shown for comparison. Arrowheads indicate the presence of EGFP foci indicative of nanocompartment formation. DIC images are shown to highlight the cell boundaries. The scale bar represents 1 μm. Representative crops of images are displayed, additional images are provided in Supplementary Figure 3.

*E. coli* C2566 cells coexpressing the shell protein EutS and the EutC^1–19^-EGFP fusion formed nanocompartments, which we observed as a single GFP focus per cell, slightly off-center or close to the pole in 85% of cells (Fig. 1, Supplementary Figure 3). This phenotype is in agreement with our previously published results^39^. A similar phenotype was apparent in construct EutJS, indicating that EutJ has no effect on EutS nanocompartment assembly (Fig. 1, Supplementary Figure 3). However, we noted a significant difference in the fluorescence signal of cells expressing EutPS. Aggregates of GFP formed at both poles in most of the cells coexpressing EutPS and EutC^1–19^-EGFP, with only a small number of cells (9%) forming nanocompartment-like foci as a third fluorescent punctate visible in the middle of the cell. The EutC^1–19^ signal sequence was required for this phenotype to be observed (Fig. 1, Supplementary Figure 3). This suggests that EutS is no longer capable of correctly forming nanocompartments in the presence of EutP. Control experiments indicate that EutP alone does not interact with EGFP or EutC^1–19^-EGFP; all cells expressing these constructs had a diffuse GFP signal (Supplementary Figure 4), negating the possibility that EutP is interacting with the cargo protein non-specifically and preventing encapsulation. Finally, cells expressing EutQS also lost the typical nanocompartment phenotype; cells were small and rounded indicating that they were stressed, and each cell had a single large aggregate of GFP close to or located at the pole of the cell (Fig. 1, Supplementary Figure 3). The same phenotype was observed when no EutS shell protein was present or when no EutC^1–19^ signal sequences was fused to EGFP, suggesting that expression of EutQ causes EGFP to aggregate (Supplementary Figure 4).

**Figure 2:**
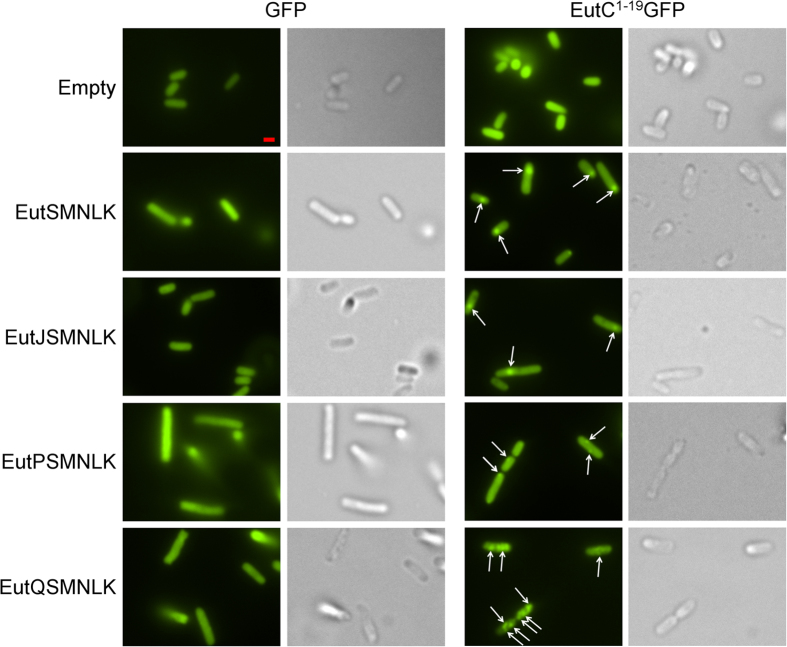
Effect of EutJ, EutP and EutQ expression on EutSMNLK nanocompartment assembly in *E. coli*. The left side of the panel displays cells co-expressing the shell proteins EutSMNLK along with EutJ, P, or Q and an untagged EGFP reporter. Images on the right side show cells co-expressing EutSMNLK along with EutJ, P, or Q and the EutC^1–19^-EGFP fusion. Control cells (Empty: expressing an empty plasmid without *eut* genes) expressing cargo proteins alone are also shown for comparison. Arrowheads indicate the presence of EGFP foci. DIC images are shown to highlight the cell boundaries. The scale bar represents 1 μm. Representative crops of images are displayed, additional images are provided in Supplementary Figure 5.

These results indicated that coexpression of EutP, and perhaps EutQ, with the single shell protein EutS was potentially disrupting or preventing correct nanocompartment assembly. To determine whether the four other shell proteins (EutMNLK) were required to facilitate correct formation of nanocompartments in the presence of EutP and EutQ, we stacked EutJ, EutP and EutQ with EutSMNLK (Supplementary Table 1). *E. coli* C2566 cells expressing EutSMNLK and EutC^1–19^-EGFP formed a single nanocompartment per cell (80% of cells), slightly off-center or close to the pole (Fig. 2, Supplementary Figure 5), as previously described^39^. EutJ had no effect on EutSMNLK nanocompartment assembly, 73% of the cells had a single nanocompartment (Fig. 2, Supplementary Figure 5). No nanocompartment-like foci were observed in cells expressing EutPSMNLK, however a few cells (19% of cells visualized) had one or two very small GFP spots localized to the cell wall (Fig. 2, Supplementary Figure 5), indicating that EutP could be preventing or disrupting EutSMNLK nanocompartment assembly. This result corroborates our findings with EutPS where large polar GFP aggregates formed, also indicating a loss of proper compartment formation. Significantly, coexpression of EutQSMNLK with EutC^1–19^-EGFP resulted in multiple GFP foci distributed across the cytoplasm throughout the entire intracellular space, in 87% of the cells visualized (Fig. 2, Supplementary Figure 5). This was in contrast to just the one or two very small GFP spots localized to the cell wall in a small percentage of the EutPSMNLK cells. These fluorescent puncta in EutQSMNLK cells were only observed when EGFP was fused to the EutC^1–19^ signal sequence, and were smaller than the single foci observed in the case of EutS or EutSMNLK without co-expression of EutQ (Figs 1 and 2). We also noted that some of the fluorescent puncta in some of the *E. coli* cells expressing EutQSMNLK appeared to be slightly unconstrained in cellular space (Supplementary Movie 1), which was in contrast to the single immobile GFP foci that we observed in *E. coli* cells expressing EutSMNLK (Supplementary Movie 2). These results pointed to EutQ potentially being responsible for multiple nanocompartment formation in a heterologous host, a finding which may have implications for understanding Eut BMC biogenesis in *S. enterica* LT2.

### EutQ may be involved in Eut BMC biogenesis in the native host *S. enterica* LT2

**Figure 3:**
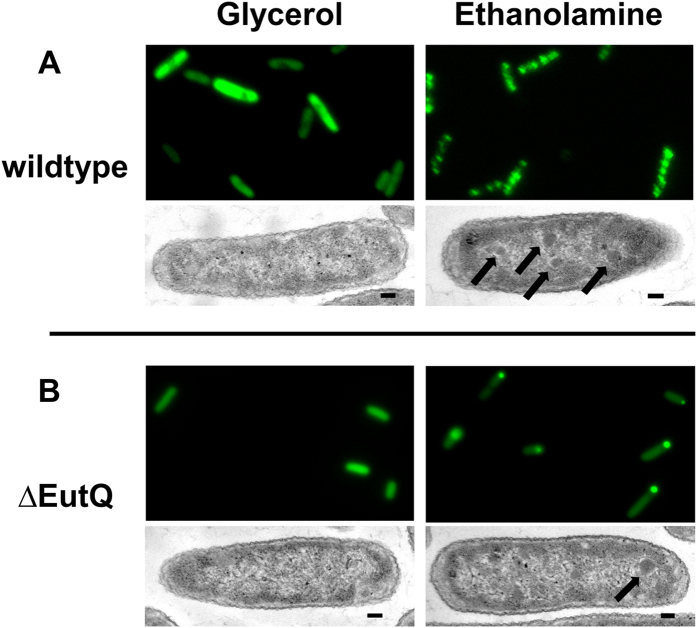
*S. enterica* LT2 ΔEutQ cells may be impaired in BMC biogenesis. (**A**) Representative cropped images (GFP fluorescence (top panels) and TEM images (bottom panels) of wild-type *S. enterica* LT2 cells transformed with EutC^1–19^-EGFP grown on either glycerol or ethanolamine. (**B**) Representative cropped images of ΔEutQ *S. enterica* LT2 grown under the same conditions. Arrowheads indicate BMC-like structures surrounded by an electron diffuse zone in TEM images. The scale bar on the TEM images represents 100 nm.

To verify whether EutQ is potentially involved in Eut BMC biogenesis and mobility, we explored the role of EutQ in the native host *S. enterica* LT2, by examining the formation of Eut BMCs in a EutQ deletion strain (strain TT24802 *eut*Q*370*∆::FRT (sw) containing a truncated version of *eut*Q with 40 bps of both the 5′ and 3′ ends of the eutQ gene remaining, kind gift of Dr. J. Roth^71^) that we label as “∆EutQ” herein. Wild type and ∆EutQ *S. enterica* LT2 cells harboring EutC^1–19^-EGFP were grown on glycerol as a control or ethanolamine to induce BMC formation, and gene transcription (or lack thereof) of EutQ and the full complement of BMC shell proteins EutSMNLK was first analyzed by RT-PCR (Supplementary Figure 6). BMC formation was subsequently analyzed *in vivo* by TEM and fluorescence microscopy (Fig. 3).

Wild type *S. enterica* LT2 does not form BMCs when grown on glycerol, as indicated by transcript analysis showing that EutQ, S, L and K are not expressed (Supplementary Figure 6) and a diffuse GFP signal (Fig. 3A). In the presence of ethanolamine, EutQ and all five shell proteins are expressed (Supplementary Figure 6); and as we have previously shown^39^, multiple unconstrained Eut BMCs are formed by *S. enterica* LT2 visualized by TEM and as multiple fluorescent puncta (Fig. 3A, Supplementary Movie 3 and time lapse imaging in^39^). These observations are similar to those seen in *E. coli* upon expression of EutQSMNLK (Fig. 2, Supplementary Movie 1).

Contrastingly, when the ∆EutQ strain was grown on ethanolamine to induce Eut BMC formation, a single immobile GFP punctum was observed at or near the pole of the cells (Fig. 3B, Supplementary Movie 4). It is not entirely clear from the fluorescent images whether these puncta represent single BMCs or also show aggregated GFP, however all five Eut BMC shell proteins are produced under these growth conditions (Supplementary Figure 6), and thin cell sectioning and TEM (Fig. 3B) revealed a single Eut BMC-like structure per cell (highlighted with arrows). These electron dense zones surrounded by an electron diffuse zone seen in Fig. 3A,B are similar to those observed by others in *S. enterica* LT2 grown on Eut BMC inducing conditions^68^, and those that we previously observed in *S. enterica* LT2 and our heterologous expression system^39^. Together these findings suggest that in contrast to the wild-type *S. enterica* LT2 strain, the ∆EutQ strain is producing a single BMC per cell, implying that EutQ could be required for the biogenesis of multiple, unconstrained BMCs in both the native host *S. enterica* LT2 and the heterologous host *E. coli*.

### Cellular structures formed during recombinant expression of EutJ, EutP, and EutQ in *E. coli*

We were surprised that EutS nanocompartments did not form correctly in the presence of EutQ, which induces multiple nanocompartment formation with EutSMNLK in our heterologous expression system. Furthermore, EutP appeared to be inhibiting correct nanocompartment formation (Fig. 2). Notably, Ras-like GTPases (like EutP) are sometimes associated with cytoskeletal elements and may form higher order structures *in vivo*^76^. Therefore, to explore whether EutQ, or either of the other proteins EutJ and EutP were forming large protein structures that could inhibit EutS nanocompartment formation, *E. coli* C2566 cells expressing all of the constructs were thin cell sectioned and visualized by TEM.

**Figure 4:**
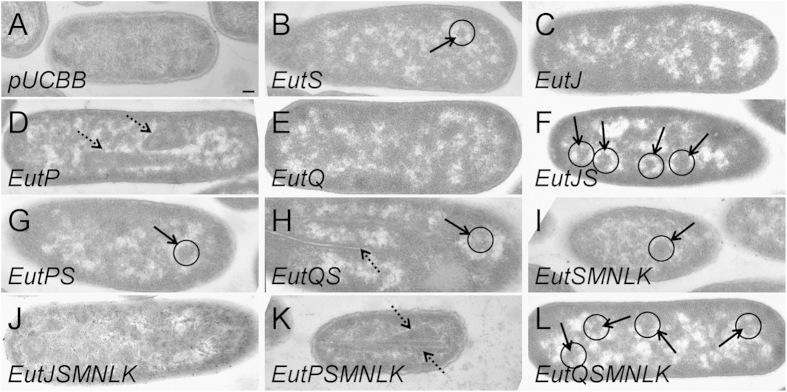
Higher order structures formed upon expression of EutJ, EutP and EutQ in *E. coli*. Subcellular structures formed in *E. coli* C2566 observed by thin sectioning and TEM. Images are as follows: *E. coli* C2566 cells expressing (**A**) empty plasmid as a control, (**B**) EutS, (**C**) EutJ, (**D**) EutP, (**E**) EutQ, (**F**) EutJS, (**G**) EutPS, (**H**) EutQS, (**I**) EutSMNLK, (**J**) EutJSMNLK, (**K**) EutPSMNLK, and (**L**) EutQSMNLK. Bold arrowheads indicate BMC-like structures, also highlighted with a circle; dashed arrowheads indicate other higher order structures. All images were taken at a magnification of x 53,000. The scale bar represents 100 nm. Representative crops of images are displayed, additional images are provided in Supplementary Figures 7–9.

Cells overexpressing EutJ did not form any visible structures (Fig. 4, Supplementary Figure 7), indicating that it does not self-assemble as a large protein complex. These findings serve to support the fact that EutJ expression has little effect on EutS or EutSMNLK assembly *in vivo* (Figs 1 and 2, Supplementary Figure 3, Supplementary Figure 5). Contrastingly, EutP self-assembled as elongated protein sheets (rectangular electron dense zones up to 500 nm in length running parallel to the axis of the cell) that also appeared folded up to give angular structures >300 nm in diameter that were offset to the axis of the cell (in 68% of cell sections) (Fig. 4, Supplementary Figure 7). It could be hypothesized that these large assemblies may potentially inhibit correct nanocompartment formation, explaining the lack of nanocompartment-like fluorescent puncta in cells expressing EutPS and EutPSMNLK (Figs 1 and 2, Supplementary Figure 3, Supplementary Figure 5). Finally, cells overexpressing EutQ did not form any visible structures (Fig. 4, Supplementary Figure 8).

To confirm that the fluorescent puncta that we observed *in vivo* (Figs 1 and 2, Supplementary Figure 3, Supplementary Figure 5) were (multiple) nanocompartments rather than random aggregation of GFP along a protein complex (like those produced by overexpression of EutP, Fig. 4, Supplementary Figure 7), we examined cells overexpressing EutJ, EutP, and EutQ with the shell proteins EutS and EutSMNLK for higher order assemblies. BMC-like structures were observed in positive control cells overexpressing the shell proteins EutS or EutSMNLK alone (visible in 32% and 36%, respectively, of cell sections) (Fig. 4, Supplementary Figures 7 and 9). The polyhedral structures typically appear as angular electron dense regions off-center in the cellular cytosol, with a diameter of 100 nm in the case of EutS and 150 nm in the case of EutSMNLK. These structures are sometimes surrounded by an electron diffuse zone, similar to what we have previously described^39^. We do not observe these structures in *E. coli* C2566 cells that are not overexpressing the shell proteins (Fig. 4, Supplementary Figure 7). Note that the percentage of cells with BMC-like structures observed by TEM is typically significantly lower than that observed by fluorescent microscope (Figs 1 and 2, Supplementary Figures 3 and 5) due to the nature of cell sectioning and the subsequent visualization procedure. Interestingly, cells overexpressing EutJS and EutJSMNLK had multiple BMC-like structures (visible in 35% and 85%, respectively, of sectioned cells) as well as other larger, rounded structures (>300 nm in diameter) and unordered electron dense regions towards the poles of cells (note the whorl-like patterns in the case of EutJSMNLK) (Fig. 4, Supplementary Figures 8 and 9), observations that contradict the fluorescence data (Figs 1 and 2, Supplementary Figures 3 and 5). The nature and role of these higher order structures is unknown. In the case of EutP, large protein sheets were no longer present upon overexpression of EutPS, and some BMC-like structures were observed (32% of sectioned cells). However, no obvious BMC-like structures were visible in cells overexpressing EutPSMNLK. Instead, large protein sheets (rectangular/angular electron dense zones that run parallel or offset to the axis of the cell) again formed (94% of sectioned cells) (Fig. 4, Supplementary Figures 8 and 9), which may explain the lack of nanocompartment formation with this construct (Figs 1 and 2, Supplementary Figures 3 and 5). Whether EutP requires the presence of EutS alone, or the other shell proteins EutMNLK to restrain uncontrolled self-assembly is not known; correct EutS nanocompartment assembly is disrupted in the presence of EutP (Fig. 1, Supplementary Figure 3). In the case of EutQ, cells overexpressing EutQS contained both BMC-like structures (35% of sectioned cells) as well as large protein tubules or filaments (long protein structures that have defined electron dense edges surrounding an electron diffuse core) (25% of sectioned cells), in some cases in the same cell. However, this phenotype was eliminated upon overexpression of EutQSMNLK. Instead, cells contained what appeared to be multiple BMC-like structures; however, these were less well defined and smaller than typical EutSMNLK BMCs (150 nm), with a diameter more similar to that of EutS BMCs (100 nm) (Fig. 4, Supplementary Figures 8 and 9). This supports our hypothesis that EutQ is involved in the formation of multiple EutSMNLK nanocompartments (Fig. 2, Supplementary Figure 5).

### Isolated nanocompartments encapsulate EutC^1–19^-EGFP

Given the propensity of EutQ to form protein tubules or filaments in the presence of EutS (Fig. 4, Supplementary Figure 8), as well as causing aggregates of the EGFP cargo protein (Supplementary Figure 4), we sought to confirm that the multiple fluorescent foci and BMC-like structure phenotype that we had observed in cells overexpressing EutQSMNLK (Figs 2 and 4, Supplementary Figures 5 and 9) were in fact related to the formation of multiple nanocompartments. Towards this aim we isolated native *S. enterica* LT2 BMCs and recombinant *E. coli* nanocompartments from cell lysates for comparative measures, and confirmed both the presence of shell proteins by mass spectrometry, as well as the structural integrity of purified compartments by negative stain TEM.

Previously, we had partially purified native BMCs from *S. enterica* LT2, and recombinant nanocompartments from *E. coli*, using an adapted sucrose gradient method^39^,^77^. We noted that BMCs appeared deflated when purified using this method^39^, which is time consuming and labor intensive (taking 2–3 days). A straightforward and rapid (taking 2–3 hours) centrifugation method for the enrichment of Pdu BMCs has recently been established by Bobik’s group^78^. We therefore chose to use this improved method for the enrichment of Eut BMCs and nanocompartments.

Initial attempts at establishing and adapting the centrifugation based method^78^ to isolate native Pdu and Eut BMCs from *S. enterica* LT2 grown on 1,2 propanediol or ethanolamine to induce BMC formation proved successful in our hands. SDS-PAGE analysis and subsequent trypsin digest and mass spectrometry (Supplementary Figures 10 and 11) of isolated BMCs confirmed the presence of metabolic enzymes and/or shell proteins associated with both types of BMC. However, it should be noted that native Eut BMCs pelleted at a lower salt concentration (250 mM KCL, as a clear glassy pellet) than that of native and heterologously expressed Pdu BMCs (500 mM KCL, as a pink glassy pellet)^41^,^78^, indicating a difference in the composition/physiochemical properties of the two different types of BMCs. The Pdu BMCs are composed of a different number of shell proteins (seven instead of the five Eut shell proteins), which may cause them to behave differently *in vitro*. Additionally, the purity of the Pdu BMCs was far superior to that of the Eut BMCs (Supplementary Figure 10), likely due to the different solubility qualities of the two protein assemblies. Furthermore, Eut BMCs isolated using this method were morphologically different to Pdu BMCs (Supplementary Figure 12). The Pdu BMCs that we isolated appear almost identical to those purified by others^78^; consistently sharp-edged polyhedral bodies with a diameter of 100 nm were observed on multiple occasions by negative stain TEM (Supplementary Figure 12A). On the other hand, Eut BMCs were fewer in number (likely due to limitations in measuring and normalizing Pdu and Eut BMC protein concentrations owing to the presence of copelleting proteins in the Eut BMC sample), appeared more round-edged, variable in size (100–250 nm in diameter), and also had a deflated appearance (Supplementary Figure 12B), again highlighting the fact that native Pdu and Eut BMCs are apparently not identical.

**Figure 5:**
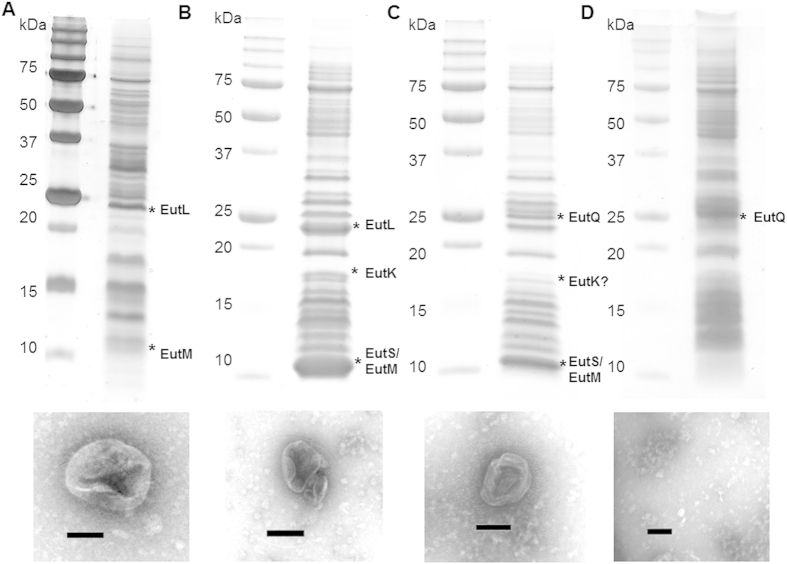
Isolated native Eut BMCs and recombinant nanocompartments. SDS-PAGE analysis of isolated native Eut BMCs from *S. enterica* LT2 and recombinant nanocompartments from *E. coli* C2566 cells expressing EutSMNLK, EutQSMNLK or EutQ. Corresponding TEM images of isolated and negatively stained polyhedral bodies are shown underneath. All samples were prepared using the centrifugation protocol described in the methods^78^. (**A**) Native Eut BMCs isolated from *S. enterica* LT2 grown on ethanolamine. (**B**) Recombinant EutSMNLK nanocompartments isolated from *E. coli* C2566. (**C**) Recombinant EutQSMNLK nanocompartments isolated from *E. coli* C2566. (**D**) Negative control, EutQ expressed in *E. coli* C2566. Bands labeled with asterisks were excised from the gel and protein identities were confirmed by LC/MS, those labeled with a question mark had peptide coverage of less than 50%. Predicted molecular weights of proteins are: EutS (11.6 kDa), EutM (9.8 kDa), EutN (10.4 kDa), EutK (17.5 kDa), EutL (22.7 kDa), EutQ (25.0 kDa). The scale bar on TEM images represents 100 nm.

Using the same method we isolated recombinant EutSMNLK nanocompartments coexpressed with EutC^1–19^-EGFP and with or without EutQ, from *E. coli* C2566. Isolated heterologously expressed nanocompartments were similar in morphology to natively expressed Eut BMCs (Fig. 5A–C), with the same rounded-edge and deflated appearance, although they were slightly smaller in size than native Eut BMCs (100–150 nm in diameter). Notably, when EutQ alone was coexpressed with EutC^1–19^-EGFP, which appears to cause aggregation of the cargo protein (Supplementary Figure 4), no BMC-like structures, filaments or large aggregates were observed by negative stain TEM (Fig. 5D), further supporting the conclusion that the polyhedral structures observed in the case of the EutSMNLK and EutQSMNLK samples (Fig. 5B+C) were nanocompartments and not aberrant aggregates or membrane fractions. The identity of the EutSMNLK shell proteins as well as EutQ in isolated nanocompartment samples was also confirmed by SDS-PAGE analysis and mass spectrometry (Fig. 5B–D).

**Figure 6:**
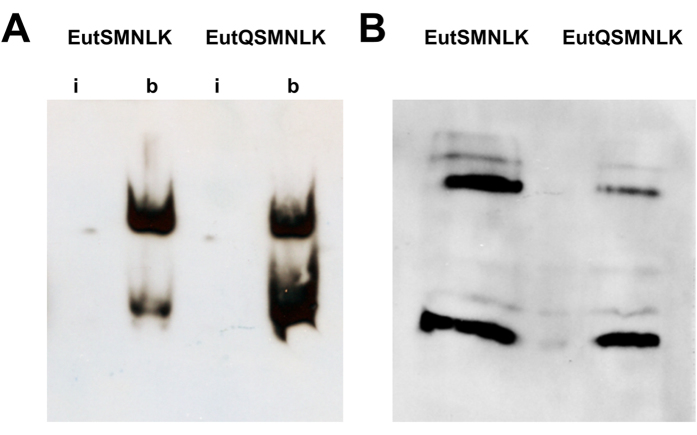
EutSMNLK and EutQSMNLK nanocompartments encapsulate cargo protein EutC^1–19^-EGFP. (**A**) Native PAGE electrophoresis and anti-GFP western blot analysis of isolated intact (i) or broken (b) nanocompartments harboring EutC^1–19^-EGFP. (**B**) SDS-PAGE electrophoresis and anti-GFP western blot analysis of broken nanocompartments harboring EutC^1–19^-EGFP reveal a multimeric assembly of cargo protein.

To confirm that the polyhedral structures observed by negative stain TEM (Fig. 5B+C) were isolated nanocompartments encapsulating EutC^1–19^-EGFP cargo protein, we conducted anti-GFP western blot analyses. Isolated EutSMNLK and EutQSMNLK nanocompartments harboring EutC^1–19^-EGFP were broken by sonication, and intact and broken shells were separated by native PAGE electrophoresis followed by exposure to anti-GFP antibody, as described previously^39^. GFP was detected when antibody was incubated with broken EutSMNLK and EutQSMNLK nanocompartments (Fig. 6A), but not when incubated with intact nanocompartments, confirming that the intact polyhedral structures (Fig. 5B+C) sequestered EutC^1–19^-EGFP within the lumen of the shell, similar to what we have previously observed^39^. GFP appeared to migrate as two separate bands on a native-PAGE gel (Fig. 6A), indicating that the cargo protein may behave as a multimeric species. When the EutSMNLK and EutQSMNLK nanocompartments encapsulating EutC^1–19^-EGFP were separated under denaturing conditions, GFP was also detected as a multimeric species (Fig. 6B), suggesting that the signal sequence-cargo protein is capable of forming stable complexes. Note, this behavior appears to relate to the presence of the EutC^1–19^ signal sequence and/or shell proteins; using the same anti-GFP western blot conditions with a EGFP standard results in the detection of a single band on native PAGE gels.

These *in vitro* data - showing that intact shells sequestering EutC^1–19^-EGFP can be isolated - unequivocally confirm that our interpretation that the fluorescent puncta we observed in cells expressing EutSMNLK and EutQSMNLK (Fig. 2, Supplementary Figure 5) is correct, they are indeed intact nanocompartments containing encapsulated EutC^1–19^-EGFP.

### Interactions between EutQ and recombinant nanocompartment shell proteins

Our results indicated that EutSMNLK nanocompartments could form correctly in the presence of EutQ (Figs 2 and 4, Supplementary Figures 5 and 9) whereas EutS nanocompartment formation was partially disrupted in the presence of EutQ (Figs 1 and 4, Supplementary Figures 3 and 8). It appeared that the presence of one or more of the shell proteins EutMNLK was required for the multiple nanocompartment phenotype. This may indicate that a shell protein/EutQ interaction is required to mediate correct nanocompartment formation, opening up the possibility that EutQ serves as an anchor point for nanocompartment biogenesis. To determine whether EutQ did interact with any of the shell proteins, we carried out *in vitro* protein pull downs.

**Figure 7:**
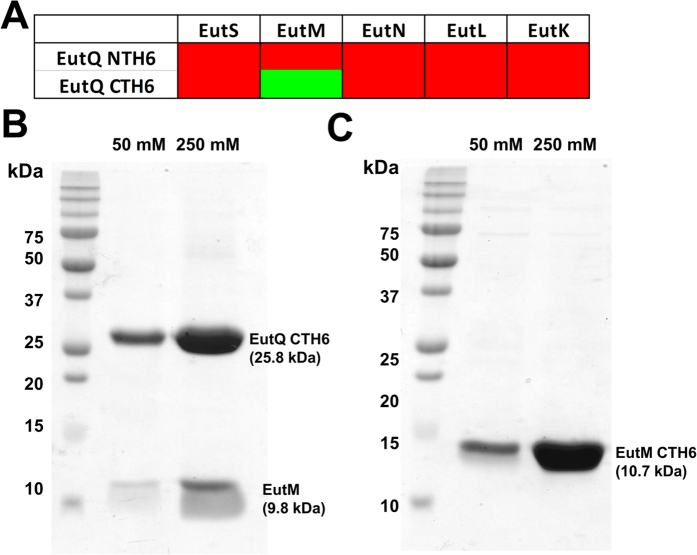
EutQ and EutM interact *in vitro*. (**A**) Green or red shaded boxes indicate a positive or negative interaction, respectively, as assessed by affinity-based pull-downs of untagged Eut shell proteins. (**B**) SDS-PAGE analysis of protein pulldowns between C-terminally His_6_ (CTH6) tagged EutQ and EutM. The first lane demonstrates the co-elution of a low level of both proteins at a low imidazole concentration (50 mM), while the second lane demonstrates co-elution of both proteins at a high imidazole concentration (250 mM). (**C**) SDS-PAGE analysis of protein pulldowns between C-terminally His_6_ (CTH6) tagged EutM and an N-terminally truncated version of EutQ (∆EutQ1–100). The first lane demonstrates the elution of a single protein at a low imidazole concentration (50 mM), while the second lane demonstrates the elution of a single protein at a high imidazole concentration (250 mM). The identity of the protein in these bands was confirmed as EutM-CTH6 by peptide mass sequencing (Supplementary Figure 13).

Using N- and C-terminally His_6_ tagged EutQ bound to a Co^2+^-affinity resin as bait, we passed *E. coli* C2566 cell lysate containing pUCBB-overexpressed untagged individual shell proteins EutS, EutM, EutN, EutL or EutK (Supplementary Table 1) over the resin as prey, similar to methods used for the Pdu shell proteins^62^. None of the shell proteins interacted with N-terminally His_6_ tagged EutQ (Fig. 7A); however, a sufficiently tight interaction between EutM and C-terminally His_6_ tagged EutQ *in vitro* resulted in co-elution of the two proteins from the resin (Fig. 7B). This may indicate that binding of the N-terminus of EutQ to the resin rendered the protein in a configuration that was not amenable to protein:protein interactions with EutM, or perhaps an interaction between the two proteins occurs close to, or at the N-terminus of EutQ. We tested the hypothesis that the N-terminus of EutQ harbored a putative interaction site for EutM by creating an N-terminally truncated version of EutQ lacking the first 100 amino acids (Supplementary Table 1), in a similar fashion to the construct design of EutQ^100–229^ *from S. enterica* LT2, the crystal structure of which has been deposited in the Protein DataBank (PDB ID: 2PYT). Truncating the N-terminus of EutQ appeared to abolish an interaction with EutM *in vitro*, with ∆EutQ1–100 remaining in the flow-through during protein pulldown experiments (Fig. 7C, Supplementary Figure 13). These findings indicate that the N-terminus of EutQ may be involved in protein interactions with EutM. It is not known what residues of EutQ mediate an interaction with EutM; no obvious surface charge distributions or hydrophobic patches that could act as potential protein interaction sites have been identified in the structurally characterized EutQ homolog CD1925 from *C. difficile* 630^25^ which lacks 27 amino acids at the N-terminus.

**Figure 8:**
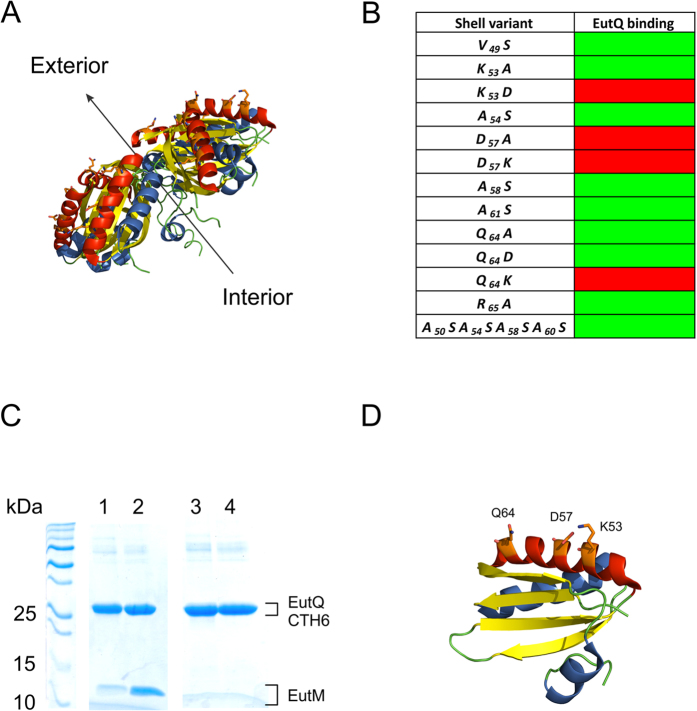
*In vitro* characterization of the putative EutQ:EutM interaction. (**A**) Crystal structure of the EutM hexamer from *E. coli* (PDB ID: 3MPW^28^). The predicted cytosol exposed helix is highlighted in red; residues predicted to be involved in electrostatic interactions with EutQ are shown as orange sticks. The arrow indicates the predicted orientation of the luminal and exterior faces of the EutM hexamer in the context of a Eut BMC shell. (**B**) Identification of EutM mutants which abolish interaction with EutQ. Green boxes indicate co-elution of EutM mutants with EutQ in pull-downs, while red boxes indicate no co-elution. (**C**) Representative SDS-PAGE analysis of a mutation that abolishes interaction between EutM and EutQ (for clarity only the D57A mutant is shown). Lanes 1 and 2 depict a control pull-down between wild-type EutM and EutQ CTH6, while lanes 3 and 4 demonstrate the loss of EutQ CTH6 binding by the EutM D57A mutant. Lanes 2 and 4 are the high imidazole (250 mM) elutions, while lanes 1 and 3 are elutions at low (50 mM) imidazole concentrations. (**D**) Cartoon representation of a EutM monomer with the residues shown to be involved in EutQ:EutM interactions highlighted as orange sticks.

We therefore turned our attention to identifying potential interaction site(s) for EutQ on the surface of EutM. Considering that EutQ appears to mediate multiple nanocompartment biogenesis (rather than be encapsulated within the lumen of the nanocompartment), we assumed that EutQ would interact with the cytosol-facing surface of EutM. A short helix has been identified on the BMC lumen-facing side of the EutM homolog PduA, which is the interaction site for the N-terminal 18 amino acid portion of the propionaldehyde dehydrogenase PduP, resulting in its encapsulation within Pdu BMCs^40^,^41^,^42^. We therefore chose to focus our search for potential interacting residues on the opposite face of EutM to that of the PduA encapsulation helix (which would correspond to α-helix 3, residues G80–F86 on EutM), using the crystal structure of EutM from *E. coli* (PDB ID: 3MPW^28^) as a guide. The putative outer face of EutM is characterized by the presence of exposed α-helix 2 (V49–Q64) on each of the EutM monomers (Fig. 8); we theorized that this would be the most suitable site for any potential protein:protein interaction. Considering that hydrophobic amino acids play a major role in interactions between PduA and PduP^40^,^41^,^42^, we mutated all hydrophobic residues along exposed α-helix 2 (V49–Q64) to polar serine (Fig. 8). EutQ co-elution with EutM was not prohibited, implying that EutQ:EutM interactions are not mediated by hydrophobic residues. Next, we explored whether electrostatic interactions were involved, by mutating all charged residues to either remove the charge or to swap the charge of the side chain. Mutants K53D, D57A, D57K and Q64K on EutM abolished EutQ:EutM interactions *in vitro*, showing that a specific electrostatic surface on the likely cytosol-exposed helix of EutM is required for EutQ binding *in vitro* (Fig. 8). Interestingly, residues K53 and D57 are well conserved across 138 EutM homologs (Supplementary Figure 14); however, not all *eut* gene clusters across different types of bacteria contain a *eut*Q gene^45^,^51^. It remains to be explored whether the interaction between EutM and EutQ (and presumably EutQ-mediated biogenesis of multiple Eut BMCs) is specific to the Eut BMC proteins from *S. enterica* LT2, or whether this is a widely conserved mechanism adopted by other bacterial species. Nonetheless, we suggest that in *S. enterica* LT2 Eut BMC and recombinant nanocompartment biogenesis, EutQ may serve as a nucleation point for EutM, potentially organizing BMCs within the cell similar to ParA organization of the carboxysome^37^,^79^, although this hypothesis remains to be verified. If this were the case, it could be envisioned that the EutQ-nucleated EutM serves as a scaffolding site for the initiation of BMC biogenesis, similar to the biogenesis of carboxysomes which requires the scaffolding protein CcmM and shell-protein recruitment protein CcmN^36^,^38^.

Finally, in an attempt to determine whether the EutQ:EutM interactions that we had observed *in vitro* were physiologically relevant, providing some support for the theories presented above, we sought to create the K53D, D57K and Q64K EutM mutants in the context of EutQSMNLK and visualize the formation (or loss) of multiple nanocompartments in *E. coli* C2566. Unfortunately, after exhaustive attempts (including the use of a tightly controlled and inducible promoter^80^ for shell protein expression) to obtain reproducible and stable phenotypes for cells expressing the EutM mutants in the context of the other four shell proteins and EutQ, we were forced to conclude that expression of these mutants in this genetic context causes severe stress for *E. coli* cells. In fact, the recombinant *E. coli* cells appeared small with large polar GFP aggregates and exhibited attenuated growth. Furthermore, sequencing of plasmids isolated from these cells showed that the mutated EutM genes were not stable, frequently accumulating additional, presumably inactivating mutations. At this point, we do not know why mutations in this surface helix of EutM cause these severe phenotypes. While these findings negate the possibility of properly testing EutQ:EutM interactions *in vivo*, they do provide us with valuable insights into the importance of levels of protein expression on heterologous nanocompartment biogenesis. Fine-tuning of individual shell protein, EutQ and cargo protein expression in *E. coli* remains to be engineered to further optimize the formation of nanocompartments for biotechnological applications.

## Discussion

The goal of this work was to identify which protein(s) encoded by the *S. enterica* LT2 *eut* operon could be involved in multiple Eut BMC biogenesis, and to express this as part of our recombinant system for the production of multiple nanocompartments in the heterologous host *E. coli* C2566. We show that overexpression of EutQ, a putative cupin domain protein of unknown function, with the shell proteins EutSMNLK can increase the number of recombinant nanocompartments produced per cell. Additionally, the nanocompartments appear to gain slight mobility upon overexpression of EutQ. While the role of EutQ in facilitating this multiple unconstrained nanocompartment phenotype is not fully understood, we hypothesize that EutQ could serve as a nucleation point for BMC biogenesis, mediated by interactions between EutQ and the shell protein EutM.

In *Synechococcus*, the cytoskeletal protein ParA self-polymerizes as filaments, and these higher order structures are required for organization of carboxysomes within the cell^79^. Biogenesis of multiple carboxysomes is initiated by aggregation of the scaffolding protein CcmM and the cargo RuBisCo along the cytoskeletal scaffold^36^,^37^ followed by CcmN-mediated recruitment of shell proteins^38^. Furthermore, the Ras-like GTPase PduV has been suggested to play a role in the spatial distribution of recombinant Pdu BMCs^62^. Members of the GTPase superfamily are involved in vesicle trafficking along the cytoskeleton^76^ and therefore it would seem logical that the PduV homolog EutP could play a similar role in Eut BMC mobility. Yet, this is not the case. Unexpectedly, overexpression of EutP disrupts nanocompartment assembly in a heterologous host.

Instead, we have shown that EutQ is involved in multiple native Eut BMC, and recombinant nanocompartment, assembly in *S. enterica* LT2 and *E. coli* C2566, respectively. How the cupin domain protein EutQ mediates this effect is not fully understood; the cupin superfamily is characterized by proteins sharing a conserved β-barrel fold, with widely diverse activities such as dioxygenase, decarboxylase, isomerase or small molecule binding^74^, but are not typically associated with cytoskeletal formation. Nonetheless, EutQ deletion from *S. enterica* LT2 results in an increased aldehyde release when cells are grown on ethanolamine^71^, and it has been suggested that EutQ is required for correct compartmentalization of EutE in Eut BMCs^73^. These findings are supported by our conclusion that EutQ is required for (multiple) Eut BMC assembly. Yet, EutQ does not appear to be absolutely essential for nanocompartment biogenesis in a heterologous host. The shell proteins EutS and EutSMNLK can self-assemble as a single nanocompartment when overexpressed in *E. coli*^39^, similar to the self-assembly of multiple compartments during overexpression of Pdu BMC^62^ and carboxysome^66^,^81^ shell proteins. However, it should be noted that neither the Pdu BMC nor the carboxysome operons appear to have a EutQ homolog. Therefore, EutQ-mediated formation of multiple BMCs may be limited to bacteria that have a EutQ-like protein; and it remains to be investigated if this is a *S. enterica* LT2 Eut BMC biogenesis specific phenomenon.

The interaction between EutQ and the shell protein EutM observed *in vitro* raises the possibility that EutQ regulates Eut BMC assembly by acting as (a) nucleation point(s) for initiating compartment growth, similar to the carboxysome biogenesis mechanism^36^,^37^,^38^. The EutM homologs PduA and CD1918 self-assemble as protein filaments when overexpressed in *E. coli*^23^,^25^ and EutM also self-assembles as protein filaments, while EutS can form nanocompartments by itself 39. Perhaps multiple EutQ:EutM aggregates can serve as shell assembly hubs, resulting in the growth of multiple nanocompartments; as opposed to a single nanocompartment whose growth could result from the propensity of EutS or EutM to self-assemble^39^.

Our long term goal is to optimize intracellular formation of multiple nanocompartments for targeted encapsulation of enzymes, with the aim of increasing multi-step pathway reaction efficiency in *E. coli* as a widely accepted recombinant host for applications biotechnology and biocatalysis. Here we have shown that it is possible to increase the number of nanocompartments produced per *E. coli* cell. Whether or not this improves overall reaction efficiency of metabolic pathways in *E. coli* remains to be explored. Nevertheless, formation of multiple compartments with encapsulated enzyme catalysts in *E. coli* should be beneficial for the production of compartments with encapsulated enzyme cargo as nanobioreactors for *in vitro* multi-enzyme biocatalytic applications.

Ultimately, targeting of multi-step pathways to (multiple) nanocompartments will likely require more than the one signal sequence that we have used to date (EutC^1–19^)^39^. Discovery and engineering of new signal sequences for targeting of multiple enzymes to our recombinant Eut nanocompartments is beyond the scope of this study, and will be addressed in future work e.g. by adopting strategies reported with Pdu BMCs^40^,^42^,^43^,^82^. Similarly, engineering of controlled, coordinated expression of both shell, accessory and cargo-proteins in non-native hosts such as *E. coli* will be required for the design of BMC nanocompartments as versatile industrially relevant platforms. Nonetheless, the work that we have presented here lays the foundation for an optimized configuration of nanocompartment assembly, which will eventually lead to the compartmentalization of pathways and biocatalytic multi-enzyme reaction cascades for the production of biotechnologically relevant compounds of choice.

## Methods

### Microbiology

*E. coli* C2566 cells were cultured aerobically in Luria-Bertani (LB) broth at 30 °C for 15 hrs with shaking at 225 rpm. *S. enterica* LT2 cells were cultured aerobically at 37 °C overnight in E medium supplemented with Vitamin B_12_ (150 nM) and either 0.2% (v/v) glycerol or 30 mM ethanolamine hydrochloride, as described elsewhere^39^,^68^. Where appropriate, cultures were supplemented with the appropriate antibiotic; ampicillin (100 μg mL^−1^), chloramphenicol (50 μg mL^−1^), or kanamycin (30 μg mL^−1^). All strains and plasmids used in this study are listed in Supplementary Table 1.

### Expression of nanocompartments in *E. coli* C2566

Recombinant nanocompartments were produced in *E. coli* C2566 cells by the overexpression of EutS or EutSMNLK using our in-house high-copy BioBrick^TM^ vector pUCBB, which contains a modified *lac* promoter allowing constitutive expression^67^. Localization of EGFP to recombinant nanocompartments in *E. coli* C2566 was achieved by coexpressing shell proteins with pACBB-EutC^1–19^-EGFP^39^. For the analysis of native Eut BMC formation in *S. enterica* LT2 wild type and ∆EutQ, cells were transformed with pBBRBB-EutC^1–19^-EGFP, and BMC production was induced by growth on ethanolamine as described above.

### Light microscopy and time-lapse imaging

Static images of *S. enterica* LT2 and *E. coli* C2566 cells were acquired using a Nikon Eclipse E800 or Nikon Eclipse 90i microscope equipped with bright field, DIC, phase and fluorescence optics including a 120 W X-Cite epi-fluorescence illuminator with blue (excitation filter 470–490 nm, barrier 520–580 nm) filter set and a 100X, 1.4 n.a. plan apo, oil immersion objective. For time-lapse imaging, 1 μL cell sample was immobilized with 10 μL of BacLight mounting oil (Life Technologies^TM^). Time-lapsed images were collected using a Nikon TiE equipped with a Lumencor SpectraX light source at 470 nm using a Plan Apo 100x, 1.45 n.a. objective with a Andor Zyla camera controlled via the Nikon Elements 4.6 software. Images were processed using 4–5 iterations of the 2D deconvolution Landweber method within Nikon Elements (module from AutoQuant^®^) to remove out-of-field fluorescence. Post-capture image analyses and cropping was conducted in Nikon NIS Elements Viewer 4.6 and GIMP 2.

### Transmission electron microscopy

*S. enterica* LT2 (grown on supplemented E medium) and *E. coli* C2566 cells (grown on LB medium) were fixed, embedded, sectioned and stained prior to TEM analysis as described previously^39^. Samples were visualized and photographed using a Philips CM12 transmission electron microscope. Post-capture alignment and cropping was conducted in GIMP 2.

### Isolation of native BMCs and recombinant nanocompartments

Enrichment of BMCs from cell pellets was conducted using methods adapted from previously published protocols^78^. Overnight cultures were harvested by centrifugation (3000 g, 30 mins) and the cell pellet was washed in Buffer A (50 mM Tris pH 8.0, 500 mM KCl, 12.5 mM MgCl_2_, 1.5% 1,2 propanediol or ethanolamine-HCl). Pellets (1 g wet weight) were resuspended in 10 mL Buffer A and cells were lysed by addition of B-PER^®^ II Bacterial Protein Extraction Reagent (60% v/v) (ThermoScientific), supplemented with lysozyme (0.25 mg mL^−1^), DTT (1 mM), PMSF (0.1 mM) and DnaseI (2 U). After 30 minutes incubation at room temperature, cell debris and supernatant were separated twice by centrifugation (9700 g, 5 mins). BMCs were pelleted from the resulting supernatant by centrifugation (43000 g, 30 mins). The small, glassy pellet was resuspended in 500–1000 μL Buffer B (50 mM Tris pH 8.0, 50 mM KCl, 5 mM MgCl_2_, 1% 1,2 propanediol or ethanolamine-HCl). Purity of BMCs was assessed by separation on a SDS-PAGE gel followed by silver stain or Coomassie stain to detect proteins, and overall protein content of the sample was determined using Pierce BCA Protein Assay Kit (Life Technologies). For TEM analysis, isolated BMC samples were diluted to 0.1 mg mL^−1^ in Buffer B. BMCs were negatively stained by applying 10 μL protein to a formvar-coated copper 200 mesh grid, and fixing in an equal volume of Trump’s fixative. Excess liquid was wicked off using filter paper, and excess salts were removed using 10 μL water. Proteins were stained in 10 μL uranyl acetate (2%) and grids were air dried prior to analysis using a Philips CM12 transmission electron microscope.

### Anti-GFP western blot analyses of encapsulated EutC^1–19^-EGFP

Isolated EutSMNLK and EutQSMNLK nanocompartments harboring EutC^1–19^-EGFP were broken by sonication. Broken and intact nanocompartments were loaded in separate lanes of a 10% native polyacrylamide gel. Following electrophoresis under non-denaturing conditions, proteins were transferred to a PVDF membrane (Roche), and GFP was detected using a HRP conjugated monoclonal primary anti-GFP antibody (Thermo Scientific). Development was conducted using the Pierce^®^ Fast Western Blot Kit with ECL substrate (Thermo Scientific). The membrane was then placed in a film cassette and was exposed to film (Bioexpress) for 2 minutes, and the film was subsequently developed.

### Trypsin digestion and peptide sequencing

Protein bands were excised from a SDS-PAGE gel and silver stain was removed from the gel slices in a 1:1 solution of potassium ferricyanide (30 mM) and sodium thiosulphate (100 mM). Proteolytic digestion with trypsin was conducted in 50 mM NH_4_HCO_3_, 5 mM CaCl_2_, 12.5 ng μL^−1^ trypsin. Peptides were extracted in acetonitrile and formic acid and were desalted prior to analysis on a LTQ mass spectrometer (ThermoScientific). Mass spectra were analyzed using PEAKS^®^7 or Scaffold version 4.2.1, with expected thresholds of 90.0% probability and at least 2 identified peptides.

## Additional Information

**How to cite this article**: Held, M. *et al.* Engineering formation of multiple recombinant Eut protein nanocompartments in *E. coli*. *Sci. Rep.* **6**, 24359; doi: 10.1038/srep24359 (2016).

## Supplementary Material

Supplementary Information

Supplementary Movie 1

Supplementary Movie 2

Supplementary Movie 3

Supplementary Movie 4
