# Supplementary material for: Engineering formation of multiple recombinant Eut protein nanocompartments in E. coli
Source: Sci Rep. 2016 Apr 11;6:24359. doi: 10.1038/srep24359 (PMC4827028; doi:10.1038/srep24359)
Supplement: Supplementary Information [file srep24359-s1.doc]

**Engineering formation of multiple recombinant Eut protein nanocompartments in *E. coli***

Mark Held, Alexander Kolb, Sarah Perdue, Szu-Yi Hsu, Sarah E. Bloch, Maureen B. Quin and Claudia Schmidt-Dannert

Department of Biochemistry, Molecular Biology and Biophysics, University of Minnesota, St. Paul, MN 55108, USA

**SUPPLEMENTARY METHODS**

**Isolation of *eut* operon ORFs and cloning.** Genomic regions encoding EutJ, EutP and EutQ were amplified from *S. enterica* LT2 DNA (ATCC #700720, Genbank #NC_003197.1) using gene-specific primers and were cloned into our in-house BioBrick^TM^ compatible vectors[^1^](#_ENREF_1). C-terminal 6X His tags were added to the end of ORFs by using the BglII and XhoI restriction sites and subsequently cloned onto our BioBrick^TM^ compatible vector pUCBB. The same process was conducted for N-terminal 6X His tags, with PCR products being digested with NdeI and NotI and cloned onto the pUCBB-NTH6 backbone. In all cases, tags and ORFs were separate by a flexible, 6X GS linker. Accession numbers for the EutJ, EutP, EutQ, EutS, EutM, EutN, EutL, EutK and EutC proteins are NP_461397.1, NP_461404.1, NP_461403.1, NP_461405.1, NP_461400.1, NP_461399.2, NP_461391.1, NP_461390.1, and NP_461392.1, respectively. Primers and plasmids used in this study are listed in **Supplementary Table 1**.

**Site-directed mutagenesis.** The creation of targeted mutations within both EutM and EutQ was achieved with the QuikChange^TM^ II site-directed mutagenesis kit (Stratagene) according to the manufacturers’ specifications. PCR products were digested with DpnI for 3 hrs to ensure adequate digestion of the parental template DNA. Colonies obtained following transformation of electrocompetent C2566 cells were sequenced to confirm the mutagenesis was successful. All primers used in this study are listed in **Supplementary Table 1**.

**Growth and transcription studies.** For growth studies on ethanolamine with *E.coli* C2566, cultures were grown in triplicate aerobically at 37 °C with shaking at 225 rpm in 500 mL E medium supplemented with 150 nM vitamin B_12_ (cyanocobalamin) and either 30 mM glycerol or 30 mM ethanolamine-HCl[^2^](#_ENREF_2), and OD_600_ of the cultures was measured at regular time points. As a control, *S. enterica* LT2 cells were grown in triplicate aerobically at 37 °C with shaking at 225 rpm in 500 mL E medium supplemented with 150 nM vitamin B_12_ (cyanocobalamin) and 30 mM ethanolamine-HCl, and OD_600_ of the cultures was measured at regular time points. For transcription studies, *E. coli* C2566 cells either harboring plasmid pUCBB EutSMNLK, or no plasmid, were grown aerobically at 37 °C with shaking at 225 rpm in 4 mL LB medium (supplemented with 100 µg mL^-1^ ampicillin in the case of pUCBB EutSMNLK) for 15 hours. A 500 µL aliquot of LB grown cells was subsequently harvested and used for RNA extraction, as described below.

**RNA extraction and reverse transcription.** RNA was stabilized and extracted using the RNAprotect® Bacteria Reagent and RNeasy® Mini Kit (Qiagen) and DNaseI treated for 1 hr (New England Biolabs) according to manufacturers‘ protocols. Reverse-transcription PCR was carried out using Qiagen‘s OneStep RT-PCR Kit according to the manufacturer‘s protocol, except reaction volumes were reduced 1/5 to a total of 10 µL, and 500 ng RNA was used per reaction. To confirm the absence of genomic DNA in RNA samples, a no-RT control was completed by heat-inactivating RTase in the master mix by incubation at 95 °C for 15 min prior to addition of RNA. Primers used in RT-PCR experiments and expected sizes of PCR products are provided in **Supplementary Table 2**.

**Preparation of cells for light microscopy.** *S. enterica* LT2 and *E. coli* C2566 cells were prepared for imaging from overnight cultures (following 15 hrs cultivation on either E medium or LB medium, respectively) by immobilization on glass coverslips (22x22 mm, 1.5 mm thickness, Corning) using 0.1 % (v/v) polyethylenimine (PEI). Cells were gently pelleted and washed with 1X phosphate-buffered saline (PBS) three times before being diluted ten-fold in 1X PBS. Coverslips were rinsed with 95 % ethanol and allowed to air dry before 200 µL of 0.1 % PEI was spread across their surface. After a 10 min incubation period, the excess PEI was removed gently by washing with Milli-Q water. The coverslips were allowed to dry completely at room temperature. Diluted cells (100 µL) were applied to the PEI-coated, coverslip surface and allowed to incubate for 30 min. The excess liquid was wicked off using a clean Kimwipe and the coverslips were inverted onto glass slides (25.4 x 76.2 mm, Corning) for immediate imaging.

**Protein pull-downs.** *E. coli* seed cultures (4 mL LB media) of all constructs were started from a single colony and were used to inoculate cultures (50 mL LB media), which were allowed to grow to saturation overnight at 37 °C with shaking at 250 rpm (note that all constructs were expressed using our in-house constitutive plasmid system pUCBB[^1^](#_ENREF_1)). For EutL and EutK, the culture volume was increased to 250 mL due to low expression levels of these proteins. All subsequent steps were conducted on or at 4 °C unless otherwise stated. After overnight growth, cells were pelleted at 3,000 g for 30 min and thoroughly resuspended in 10 mL of Buffer A (50 mM Tris-HCl pH 8.0, 250 mM NaCl, 5 mM imidazole and 1 mM phenylmethanesulfonyl fluoride (PMSF)). Cells were sonicated (Branson Digital Sonifier 450) at 30 % amplitude for 4 min total at a frequency of 1 sec on, 2 sec off. Lysed cells were immediately centrifuged at 9,700 g for 30 min. A 50 µL aliquot of the supernatant was collected at this time. A 1 mL resin bed of cobalt-charged TALON resin (ClonTech) was prepared by centrifuging 2 mL of resin in a 15 mL conical tube at 700 g for 2 min, followed by a 10-fold volume wash with Buffer A. This process was repeated three times. Supernatants containing His-tagged bait proteins were added to the resin and allowed to bind for 1 hr. Following binding, the resin was pelleted at 700 g for 5 min, followed by a 10 mL wash with Buffer A. This process was repeated three times, with 50 µL aliquots collected after the first and final washes. Prey proteins were prepared as described above and added to the resin containing bait proteins previously bound to it. Prey proteins were allowed to bind for 1 hr, with excess protein being washed off the resin as described above. Following prey binding, the resin was re-suspended in 5 mL of Buffer A and applied to empty gravity-flow columns (BioRad, 1.5 x 12 cm). After settling, the 5 mL of Buffer A was allowed to pass through the column. Bound proteins were eluted from the column using 5 mL of either Buffer B or Buffer C (identical to Buffer A except they contain no PMSF and contain either 50 mM or 250 mM imidazole for Buffer B and C, respectively). For SDS-PAGE analysis, samples were added 1:1 (v/v) to a standard 2X SDS loading buffer and boiled for 15 min before loading onto 6-18 % precast, gradient TGX^TM^ SDS gels (BioRad) or gels made in-house (8 % stacking, 15 % resolving gel) and separated for 40 min at 240 V alongside the Precision Plus^TM^ SDS protein ladder (BioRad). Staining was achieved using BioSafe® Coomassie stain (BioRad) for 20 min at room temperature.

**Consensus and structural modelling.** EutM homologs were identified using BLASTp[^3^](#_ENREF_3), using the BLOSUM62 matrix, expected threshold 10 and max target sequences 250 as search parameters. The list of homologs was manually curated for redundancies and incomplete sequences, leaving 138 unique sequences. It was manually confirmed that this list contained EutM homologs from bacterial species that do contain a *eut*Q gene in their putative *eut* gene clusters[^4^](#_ENREF_4), as well as proteins annotated as PduA as outliers. The curated list was used in a ClustalW[^5^](#_ENREF_5) alignment (BLOSUM matrix, gap opening penalty 10) in MEGA 6[^6^](#_ENREF_6). The degree of conservation across homologs was assessed using the WebLogo[^7^](#_ENREF_7) server. All analyses of published crystal structures for EutM (PDB ID: 3MPW[^8^](#_ENREF_8)) and EutQ (PDB ID: 2PYT and 4AXO[^9^](#_ENREF_9)) were conducted using PyMOL (Schrödinger, LLC).

**SUPPLEMENTARY TABLES AND FIGURES**

**Supplementary Table 1. Strains, plasmids and primers used in this study.**

| **Strain** | **Reference/Source** |
| --- | --- |
| *E. coli* C2566 | New England BioLabs |
| *S. enterica* LT2 | ATCC # 700720 |
| *S. enterica* LT2 TT24802 (*eutQ370*∆::FRT (sw)) | [^10^](#_ENREF_10) |
|  |  |
|  |  |
| **Plasmid** | **Reference** |
| pACBB EGFP | [^1^](#_ENREF_1) |
| pBBRBB EGFP | [^1^](#_ENREF_1) |
| pUCBB EutS | [^11^](#_ENREF_11) |
| pUCBB EutSMNLK | [^11^](#_ENREF_11) |
| pACBB EutC^1-19^-EGFP | [^11^](#_ENREF_11) |
| pBBRBB EutC^1-19^-EGFP | [^11^](#_ENREF_11) |
| pUCBB EutJ | This study |
| pUCBB EutP | This study |
| pUCBB EutQ | This study |
| pUCBB EutJS | This study |
| pUCBB EutPS | This study |
| pUCBB EutQS | This study |
| pUCBB EutJSMNLK | This study |
| pUCBB EutPSMNLK | This study |
| pUCBB EutQSMNLK | This study |
| pUCBB EutQ NTH6 | This study |
| pUCBB EutQ CTH6 | This study |
| pUCBB EutM V49S | This study |
| pUCBB EutM K53A | This study |
| pUCBB EutM K53D | This study |
| pUCBB EutM A54S | This study |
| pUCBB EutM D57A | This study |
| pUCBB EutM D57K | This study |
| pUCBB EutM A58S | This study |
| pUCBB EutM A61S | This study |
| pUCBB EutM Q64A | This study |
| pUCBB EutM Q64D | This study |
| pUCBB EutM Q64K | This study |
| pUCBB EutM A50S A54S A58S A61S | This study |
| pUCBB EutQ E64A | This study |
| pUCBB EutQ R67A | This study |
| pUCBB EutQ E91A | This study |
| pUCBB EutQ K95A | This study |
| pUCBB EutQΔ1-100 | This study |
| pUCBB EutS-mCherry-MNLK | This study |
|  |  |
|  |  |
| **Primer name** | **Sequence 5' - 3'** |
| Eut J BglII F | agatctATGGCGCACGACGAACAACTC |
| EutJ NotI R | gcggccgcTCAGCTTGCATAGAGTCCCTCC |
| EutP BglII F | agatctATGAAACGTATTGCTTTTGTC |
| EutP NotI R | gcggccgcTTAGCTGTGATAAGTTTTTTC |
| EutQ BglII F | agatctGTGAAAAAACTTATCACAGC |
| EutQ NotI R | gcggccgcTCATACGGATTGCCAGTTTGC |
| EutQ gDNA SeqF | GACATATCGCCGTTATCAGC |
| EutQ gDNA SeqR | ACTTAATGCGCAGACGGCGAC |
| EutM A50S F | TGGCGATGTGAGCGCGTGCAAAG |
| EutM A50S R | CTTTGCACGCGCTCACATCGCCA |
| EutM V49A F | GCGTGGCGATGCGGCGGCGTGCA |
| EutM V49A R | TGCACGCCGCCGCATCGCCACGC |
| EutM K53A F | ATGTGGCGGCGTGCGCAGCCGCAACCGATG |
| EutM K53A R | CATCGGTTGCGGCTGCGCACGCCGCCACAT |
| EutM A54S F | GTGGCGGCGTGCAAATCCGCAACCGA |
| EutM A54S R | TCGGTTGCGGATTTGCACGCCGCCAC |
| EutM D57A F | AAAGCCGCAACCGCGGCTGGCGCCGCTG |
| EutM D57A R | CAGCGGCGCCAGCCGCGGTTGCGGCTTT |
| EutM A58S F | CAAAGCCGCAACCGATTCTGGCGCCG |
| EutM A58S R | CGGCGCCAGAATCGGTTGCGGCTTTG |
| EutM A60S F | CGATGCTGGCAGCGCTGCGGCGC |
| EutM A60S R | GCGCCGCAGCGCTGCCAGCATCG |
| EutM A61S F | ATGCTGGCGCCTCTGCGGCGCAG |
| EutM A61S R | CTGCGCCGCAGAGGCGCCAGCAT |
| EutM Q64A F | GCCGCTGCGGCGGCGCGCATTGGCGA |
| EutM Q64A R | TCGCCAATGCGCGCCGCCGCAGCGGC |
| EutM A54S F | GTGGCGGCGTGCAAATCCGCAACCGA |
| EutM A54S R | TCGGTTGCGGATTTGCACGCCGCCAC |
| EutM K53D F | GATGTGGCGGCGTGCGATGCCGCAACCGATGCT |
| EutM K53D R | AGCATCGGTTGCGGCATCGCACGCCGCCACATC |
| EutM D57K F | TGTGGCGGCGTGCAAAGCCGCAACCAAAGCTGGCGCCGCTGCGGCGCAGCGCATT |
| EutM D57K R | CAATGCGCTGCGCCGCAGCGGCGCCAGCTTTGGTTGCGGCTTTGCACGCCGCCACA |
| EutM Q64D F | TGGCGCCGCTGCGGCGGATCGCATTGGCGAGTTGG |
| EutM Q64D R | CCAACTCGCCAATGCGATCCGCCGCAGCGGCGCCA |
| EutM Q64K F | TGGCGCCGCTGCGGCGAAACGCATTGGCGAGTTGG |
| EutM Q64K R | CCAACTCGCCAATGCGTTTCGCCGCAGCGGCGCCA |
| EutM R65A F | TGCGGCGCAGGCGATTGGCGAGT |
| EutM R65A R | ACTCGCCAATCGCCTGCGCCGCA |
| EutM A50S-A54S F | GTGAGCGCGTGCAAATCCGCAACCGATGCTG |
| EutM A50S-A54S R | CAGCATCGGTTGCGGATTTGCACGCGCTCAC |
| EutM A50S-A54S-A58S F | AAATCCGCAACCGATTCTGGCGCCGCTGCGG |
| EutM A50S-A54S-A58S R | CCGCAGCGGCGCCAGAATCGGTTGCGGATTT |
| EutM A50S-A54S-A58S-A60S F | GCAACCGATTCTGGCAGCGCTGCGGCGCAGCG |
| EutM A50S-A54S-A58S-A60S R | CGCTGCGCCGCAGCGCTGCCAGAATCGGTTGC |
| EutQ E64A F | CATCGGCGCAGGCGTGTAAAAGCGcgAGCCAGCGCATTCGGGAAGCCATC |
| EutQ E64A R | GATGGCTTCCCGAATGCGCTGGCTcgCGCTTTTACACGCCTGCGCCGATG |
| EutQ R67A F | CAGGCGTGTAAAAGCGAAAGCCAGgcgATTCGGGAAGCCATCATCGCGCAG |
| EutQ R67A R | CTGCGCGATGATGGCTTCCCGAATcgcCTGGCTTTCGCTTTTACACGCCTG |
| EutQ E91A F | GAAAGCCTGGTGGCGCAGTTGATGGcgAAAGTGCTGAAGGAAAAGCAGTC |
| EutQ E91A R | GACTGCTTTTCCTTCAGCACTTTcgCCATCAACTGCGCCACCAGGCTTTC |
| EutQ K95A F | GCGCAGTTGATGGAAAAAGTGCTGgcGGAAAAGCAGTCGCTGGAACTGGG |
| EutQ K95A R | CCCAGTTCCAGCGACTGCTTTTCCgcCAGCACTTTTTCCATCAACTGCGC |
| EutQ NTH6-NdeI-GS-F | catatgGGCTCTGGCTCTGGCTCTGGCTCTGGCTCTGGCTCTAAAAAACTTATCACAGCTAAC |
| EutQ-GS-XhoI-CTH6-R | ctcgagAGAGCCAGAGCCAGAGCCAGAGCCAGAGCCAGAGCCTACGGATTGCCAGTTTGCAGG |
| EutM NTH6-NdeI-GS-F | catatgGGCTCTGGCTCTGGCTCTGGCTCTGGCTCTGGCTCTGAAGCATTAGGAATGATTGAAAC |
| EutM-GS-XhoI-CTH6-R | ctcgagAGAGCCAGAGCCAGAGCCAGAGCCAGAGCCAGAGCCAATGTTGCTGTCGCCTTTG |

**Supplementary Table 2. Primers used in RT-PCR experiments and expected sizes of genes**

| **Gene name** | **Primer sequence** | **Expected size of gene (bp)** |
| --- | --- | --- |
| rsmA | rsmA_FWD: ATGAATAATCGAGTCCACCAGGGC  rsmA_REV: TTAACTCTCCTGCAAAGGCGCGT | 822 |
| eutS | eutS_FWD: ATGGATAAAGAACGCATCATTCAGGAATTTGTG  eutS_REV: TTAACTTTTGGTCATTTCACAAAGCG | 336 |
| eutM | eutM_FWD: ATGGAAGCATTAGGAATGATCGAAACCC  eutM_REV: TTACAGGTTGCTGCTATCGCCTTTCAGG | 294 |
| eutN | eutN_FWD: ATGAAACTGGCAGTCGTCACTGG  eutN_REV: TTATTTGTGGAAAATTACCTGACCGC | 288 |
| eutL | eutL_FWD: ATGCCAGCTTTAGATTTGATTCGAC  eutL_REV: TTACGCACGCTGGATTGGATTAC | 660 |
| eutK | eutK_FWD: ATGATCAATGCACTGGGATTGCTG  eutK_REV: TTAATGGGGCTTGAGACGATAAC | 501 |
| eutQ | eutQ_FWD: GTGAAAAAACTTATCACAGC  eutQ_REV: TCATACGGATTGCCAGTTTGC | 690 |

**
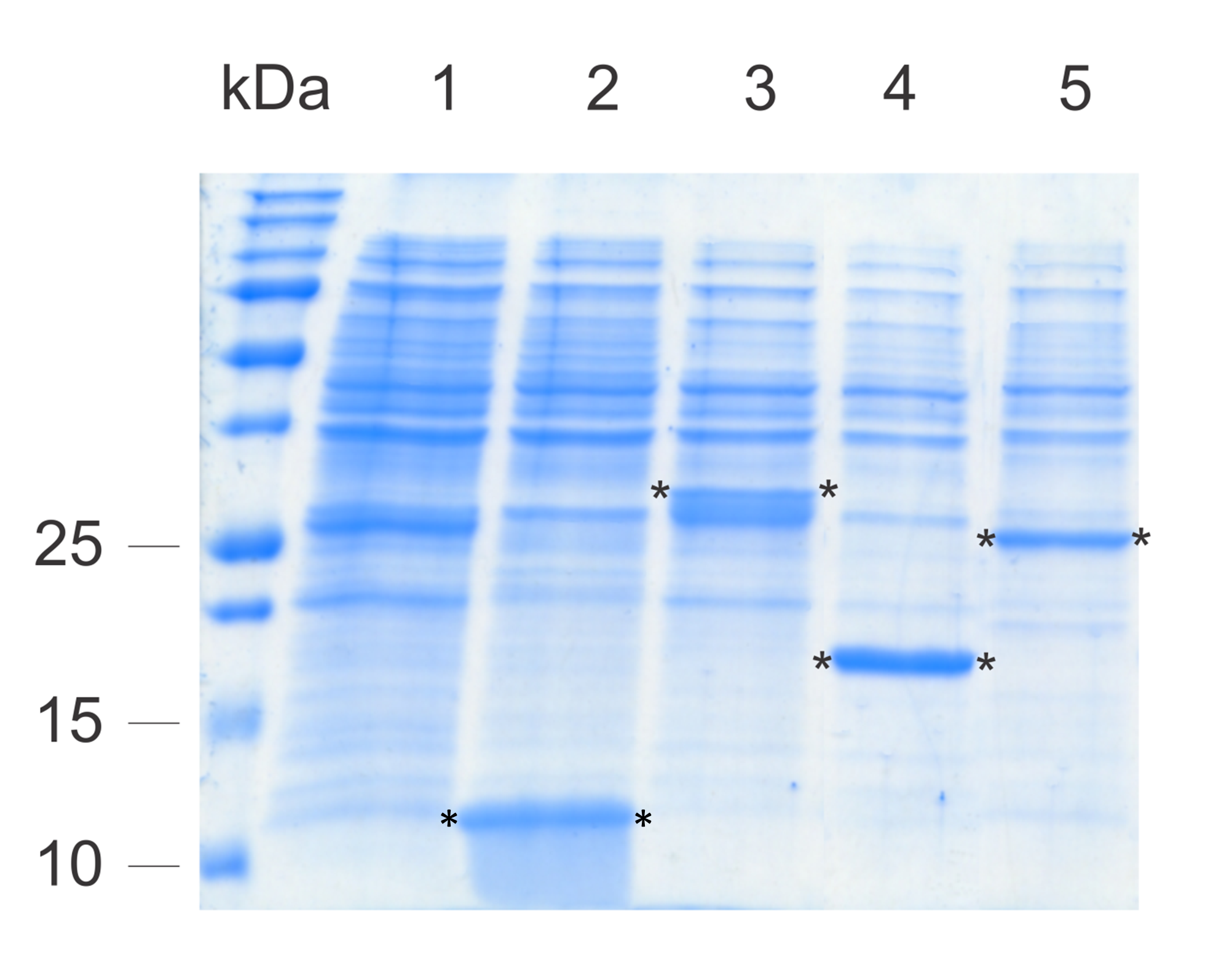
**

**Supplementary Figure 1. Expression of EutJ, EutP and EutQ in *E. coli* C2566.** Soluble protein fractions from *E. coli* C2566 cultures expressing EutJ, EutP or EutQ. Control cultures expressing EGFP alone and the Eut BMC shell protein EutS are included for reference. Lane 1 – EGFP; Lane 2 – EutS; Lane 3 – EutJ; Lane 4 – EutP; Lane 5 – EutQ; kDa – marker. The predicted molecular weight of each protein is 26, 11, 27, 18 and 25 kDa for EGFP, EutS, EutJ, EutP and EutQ, respectively. Bands corresponding to EutS, EutJ, EutP and EutQ are marked with asterisks.


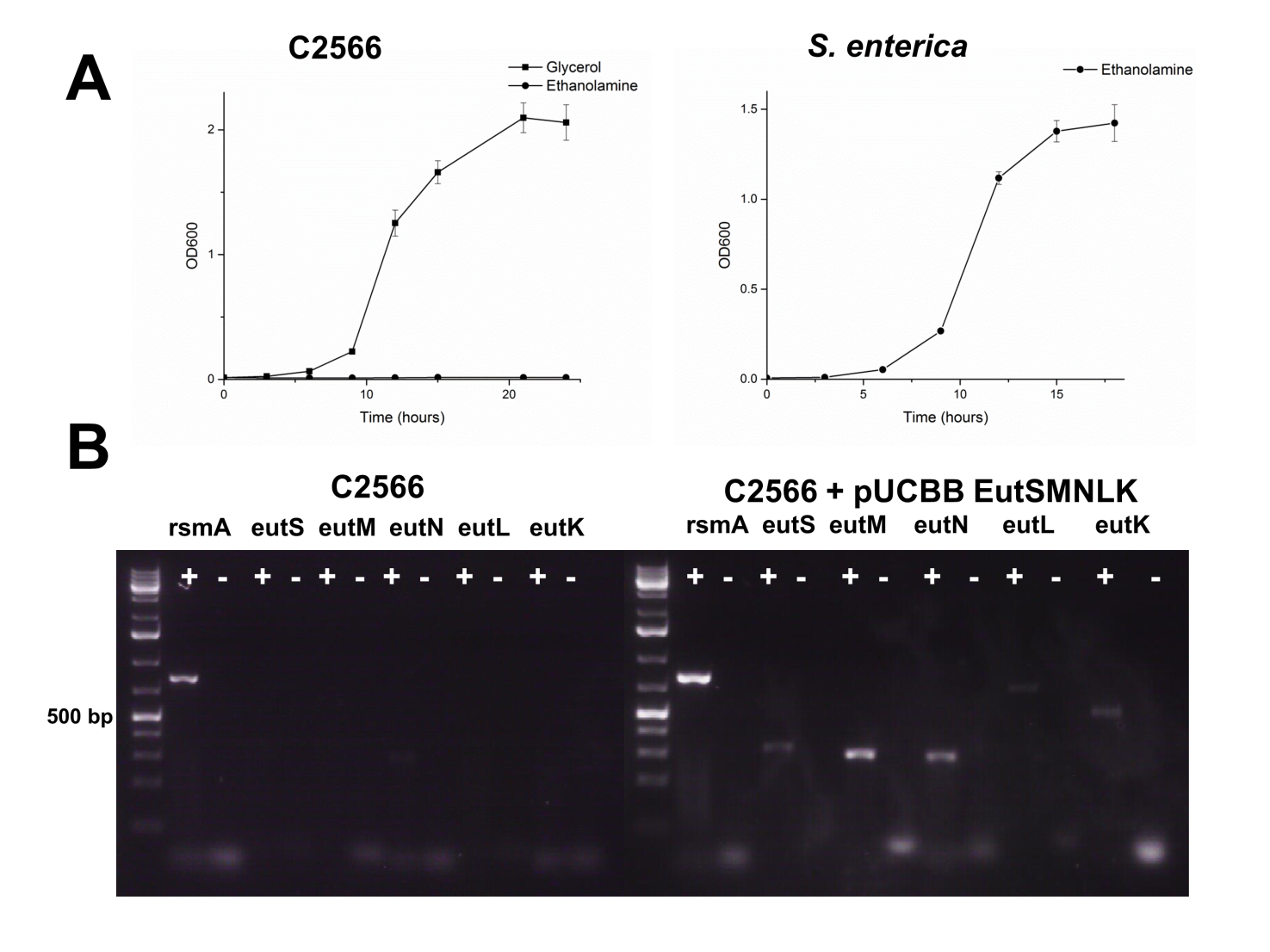


**Supplementary Figure 2. *E.coli* C2566 does not form BMCs** **because the *eut* operon is non functional** (**A**) For *E. coli* C2566, glycerol is a suitable carbon source to sustain growth, however, no growth is observed on ethanolamine, suggesting that this strain lacks a functional *eut* operon and is incapable of producing BMCs. *S. enterica* LT2 does produce BMCs and is capable of growth on ethanolamine. (**B**) RT-PCR shows transcript levels of *eut* genes in *E. coli* C2566. The gene encoding the posttranscriptional regulator rsmA serves as a positive control. Transcription of the genes encoding the five Eut BMC shell proteins in *E. coli* C2566 cells either harboring no plasmid, or harboring plasmid pUCBB EutSMNLK was tested. Cells were grown under lab standard conditions in LB medium. Lanes labeled + have active reverse transcriptase, lanes labeled – lack active reverse transcriptase, and serve as a negative control to show that there is no contaminating genomic DNA present. The genes encoding Eut shell proteins are not expressed under standard lab conditions in *E. coli* C2566, confirming that the *eut* operon is non functional in this lab strain.


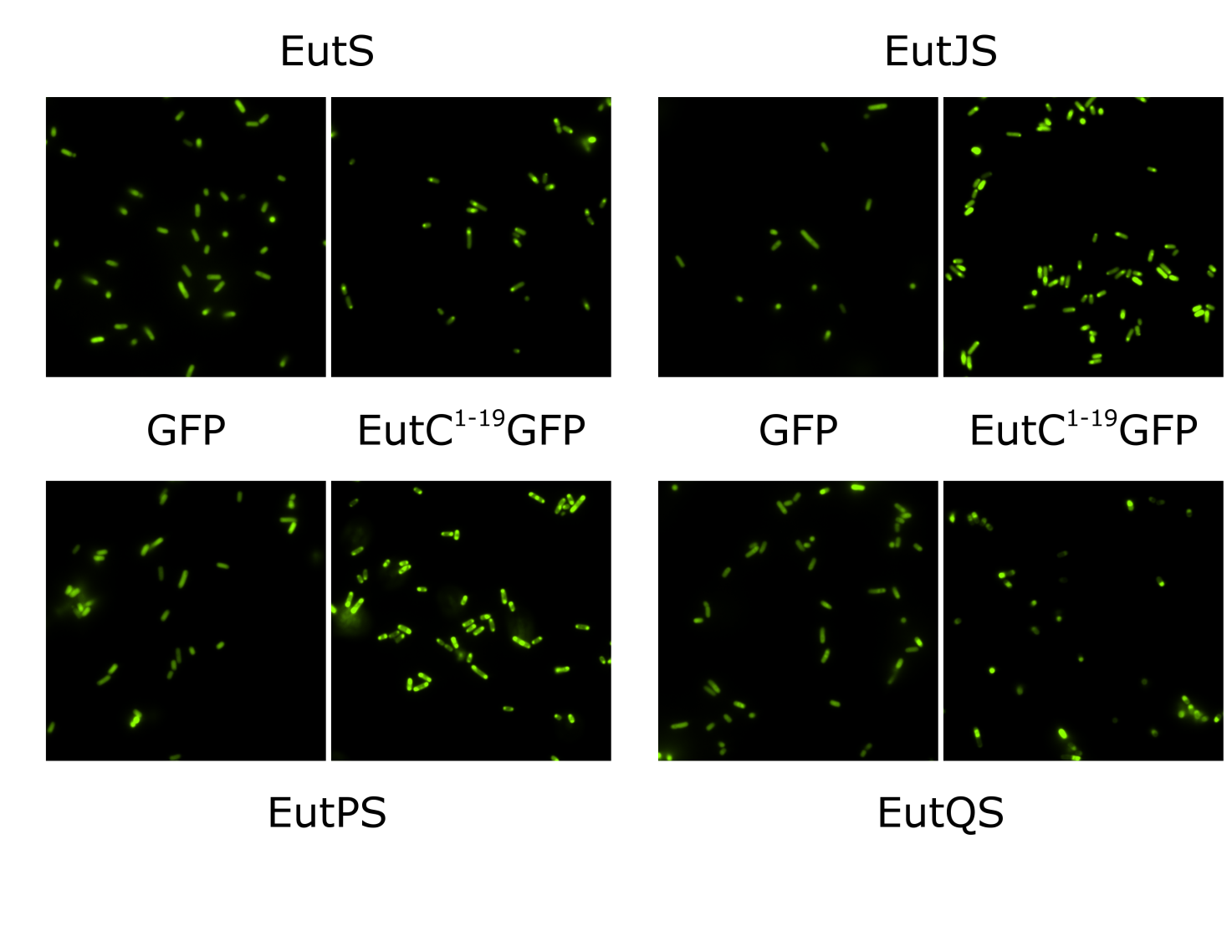


**Supplementary Figure 3. Effect of EutJ, EutP and EutQ expression on EutS nanocompartment assembly in *E. coli*.** These additional images, which show a wider field of view with a larger number of cells visible, are provided to support **Figure 1**.


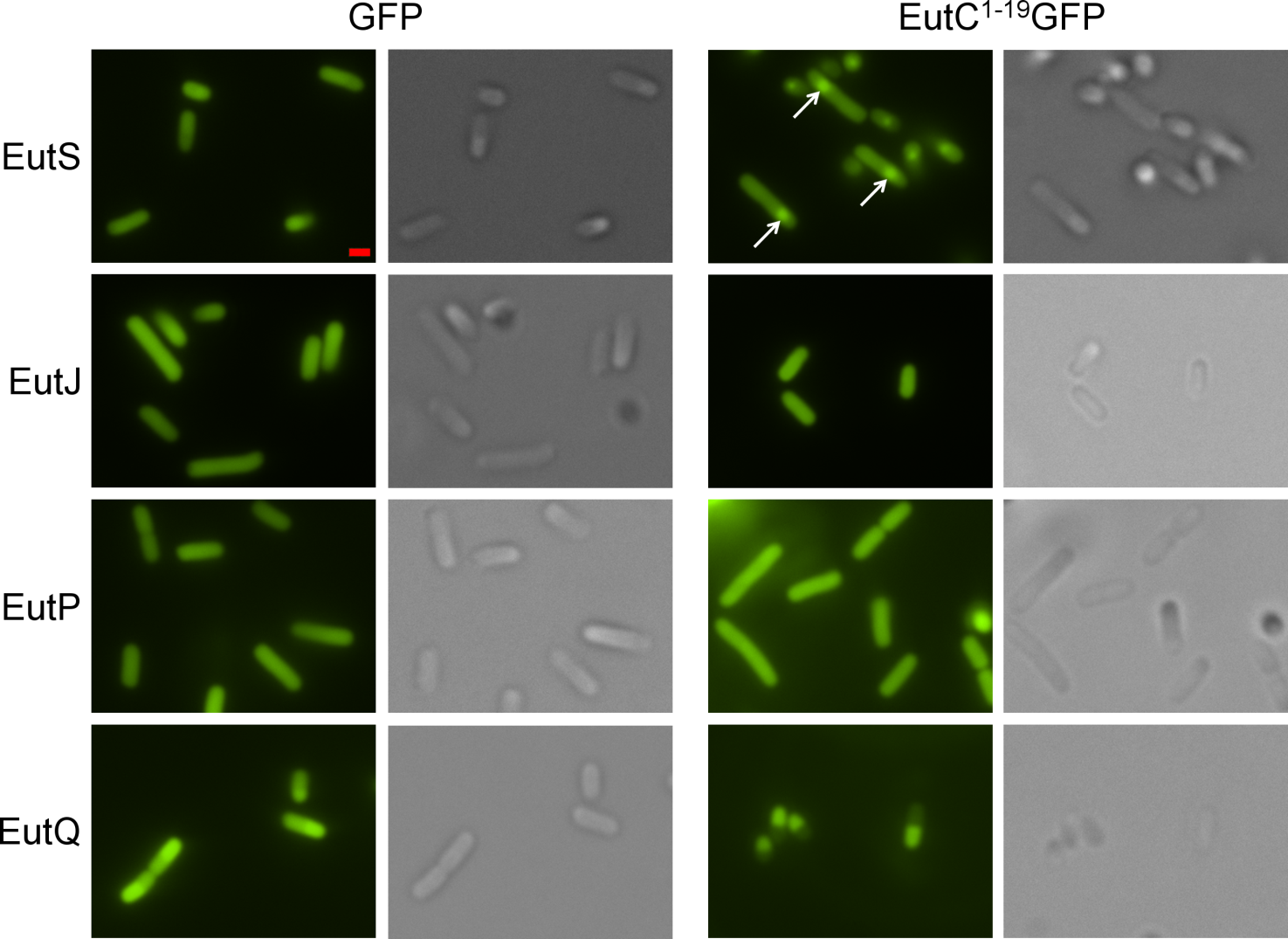


**Supplementary Figure 4. Co-expression of EGFP cargo proteins with EutS, EutJ, EutP and EutQ in *E. coli*.** EutJ, EutP, or EutQ were coexpressed with EGFP or the EutC^1-19^-EGFP fusion. EutS, which is necessary and sufficient for recombinant nanocompartment formation in *E. coli*, is included as a positive control (top panel, nanocompartments indicated by arrowheads). The scale bar represents 1 µm. Representative crops of images are displayed. DIC images are shown to highlight the cell boundaries.

**
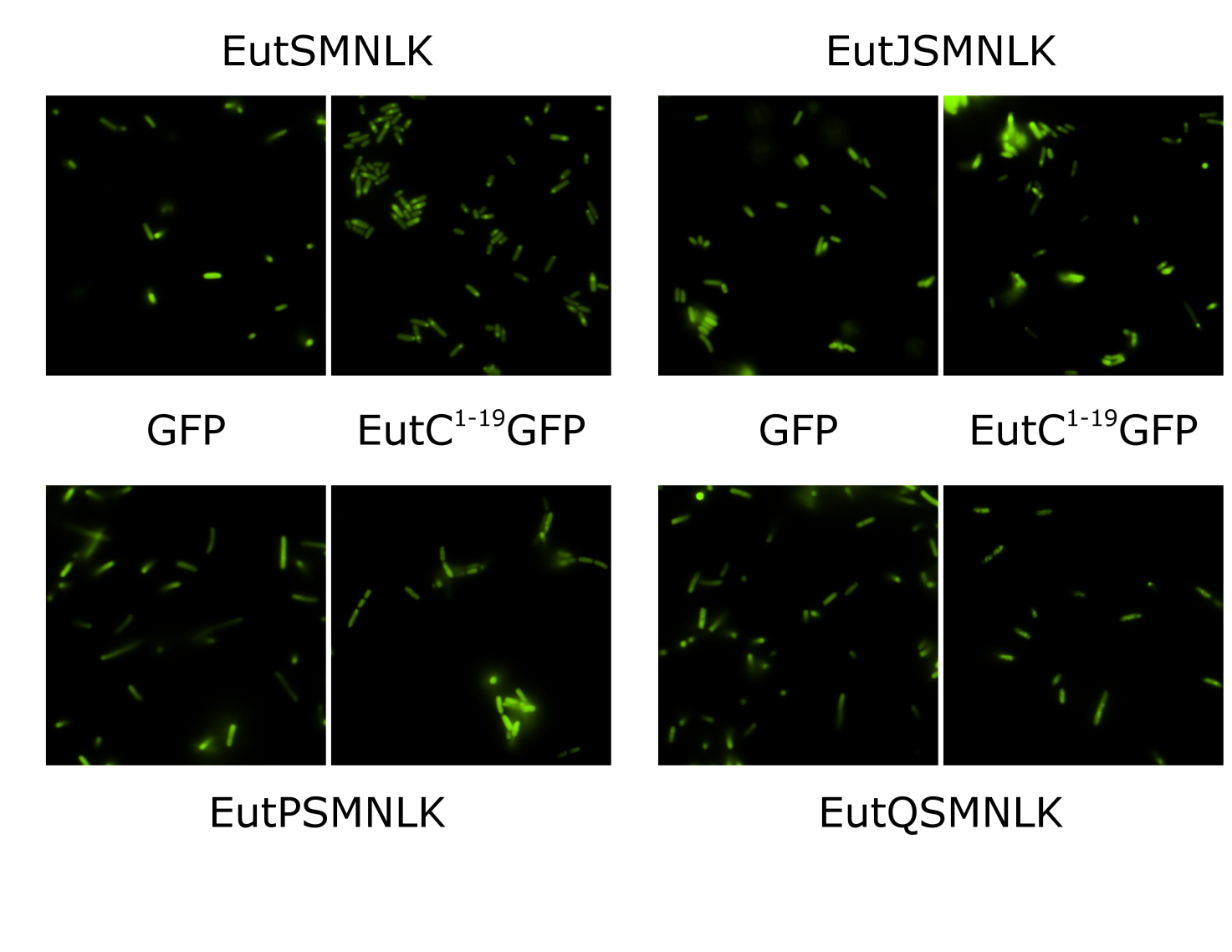
**

**Supplementary Figure 5. Effect of EutJ, EutP and EutQ expression on EutSMNLK nanocompartment assembly in *E. coli.*** These additional images, which show a wider field of view with a larger number of cells visible, are provided to support **Figure 2.**


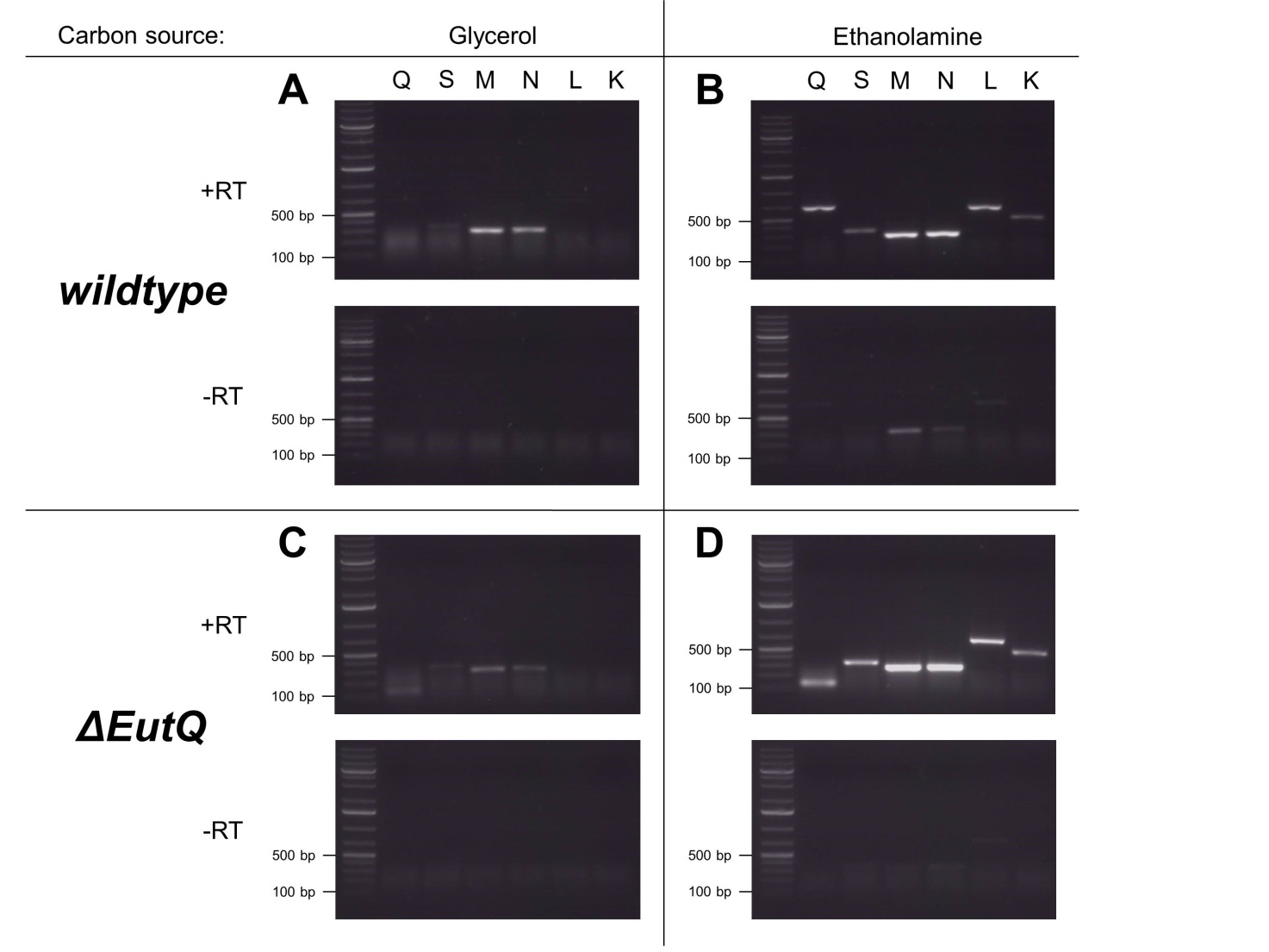
**Supplementary Figure 6. The *eutQ* knockout does not affect expression of other, proximal *eut* operon genes.** Wild type and *ΔEutQ* *S. enterica* LT2 cells were cultured in minimal media at 30 °C for 15hrs, after which RNA was stabilized, extracted, and subjected to reverse transcription PCR. Letters refer to the open reading frames of the corresponding *eut* operon genes. +RT; active reverse transcriptase, -RT; control lacking reverse transcriptase. When cultured on glycerol, both strains show a low-level of EutS, EutM and EutN transcripts (**A, C**). When cultured using ethanolamine as the sole carbon source, wild-type cells show high levels of transcripts for full-length EutQ and all five shell proteins (**B**). EutQ knockout cells show the presence of the truncated EutQ transcript and all five shell proteins at levels similar to wild-type (**D**).


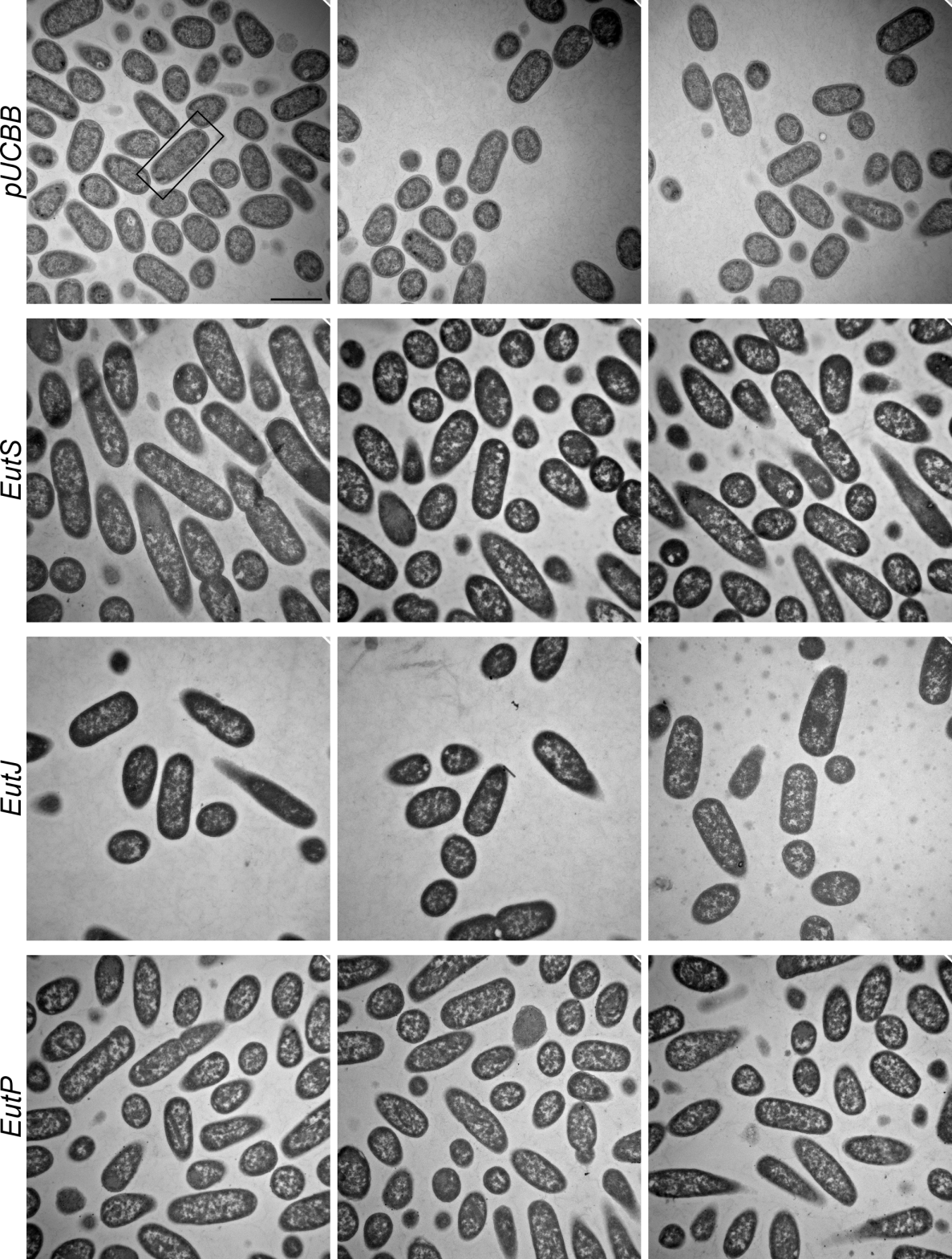


**Supplementary Figure 7. Higher order structures formed upon expression of empty plasmid, EutS, EutJ and EutP in *E. coli*.** These additional images, which were taken at a magnification of x 19,000, show a wider field of view with a larger number of cells visible, and are provided to support **Figure 4**. Where possible, cells imaged in **Figure 4** are highlighted by a box. The scale bar represents 1 µm.


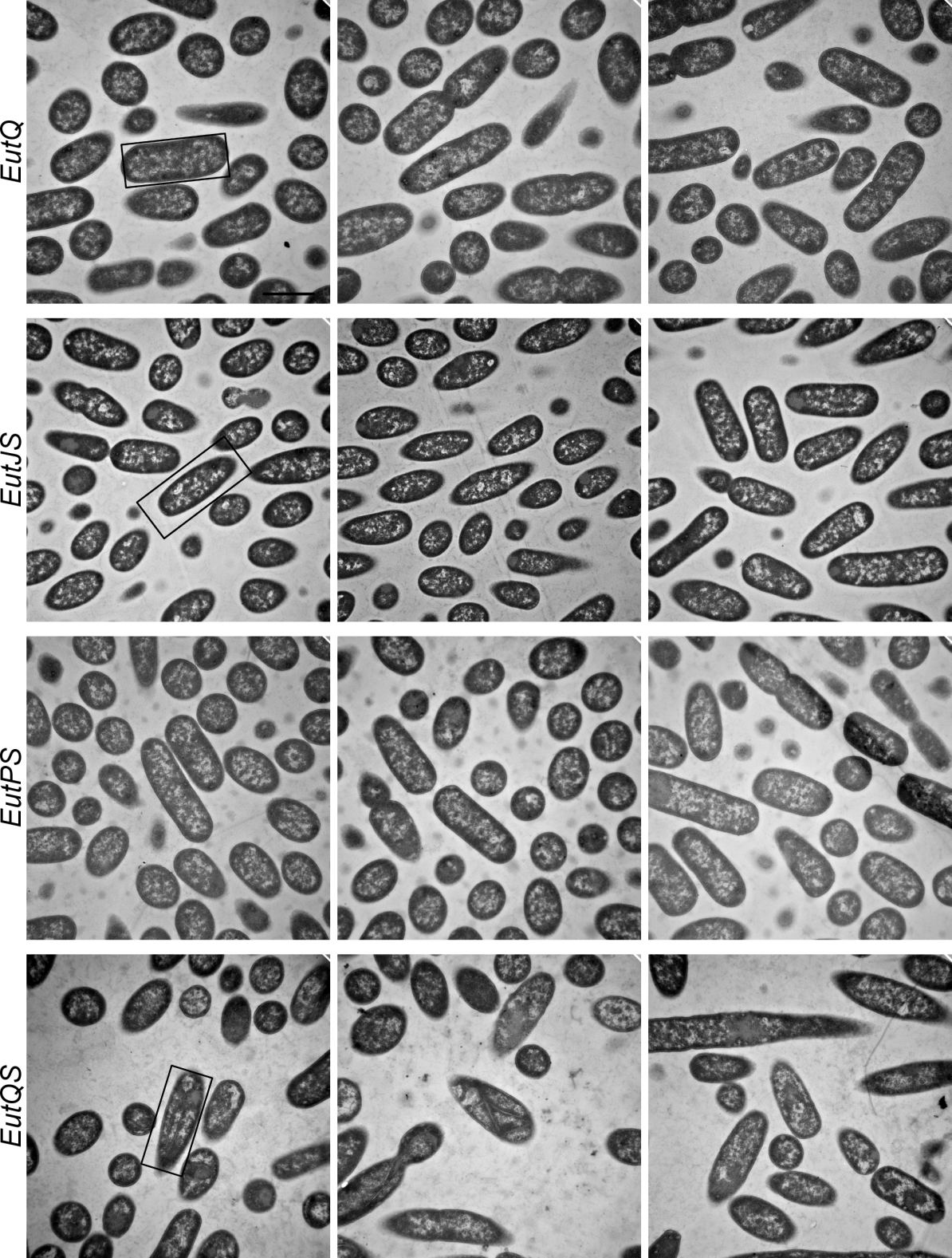


**Supplementary Figure 8. Higher order structures formed upon expression of EutQ, EutJS, EutPS and EutQS in *E. coli*.** These additional images, which were taken at a magnification of x 19,000, show a wider field of view with a larger number of cells visible, and are provided to support **Figure 4**. Where possible, cells imaged in **Figure 4** are highlighted by a box. The scale bar represents 1 µm.


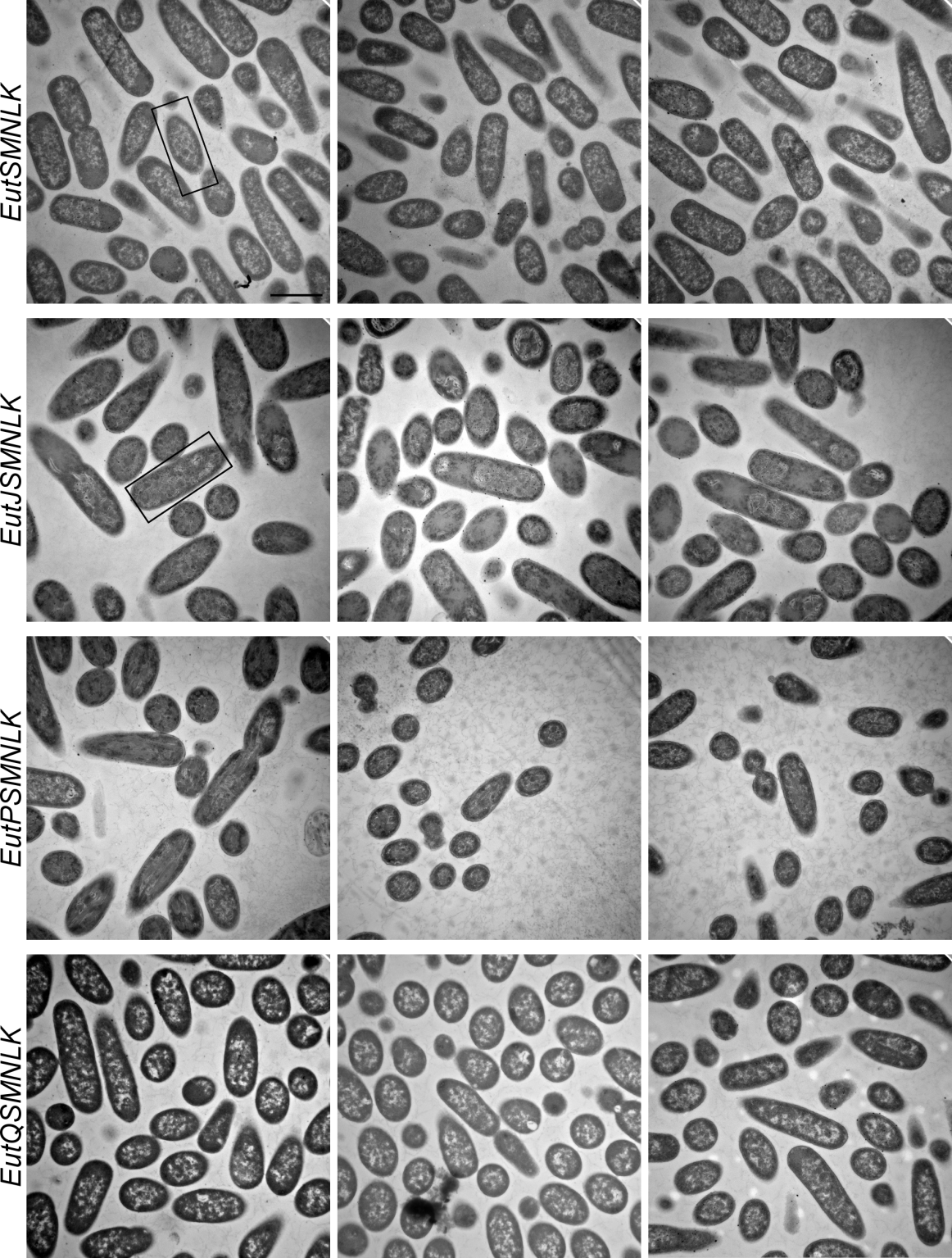


**Supplementary Figure 9. Higher order structures formed upon expression of EutSMNLK, EutJSMNLK, EutPSMNLK and EutQSMNLK in *E. coli*.** These additional images, which were taken at a magnification of x 19,000, show a wider field of view with a larger number of cells visible, and are provided to support **Figure 4**. Where possible, cells imaged in **Figure 4** are highlighted by a box. The scale bar represents 1 µm.


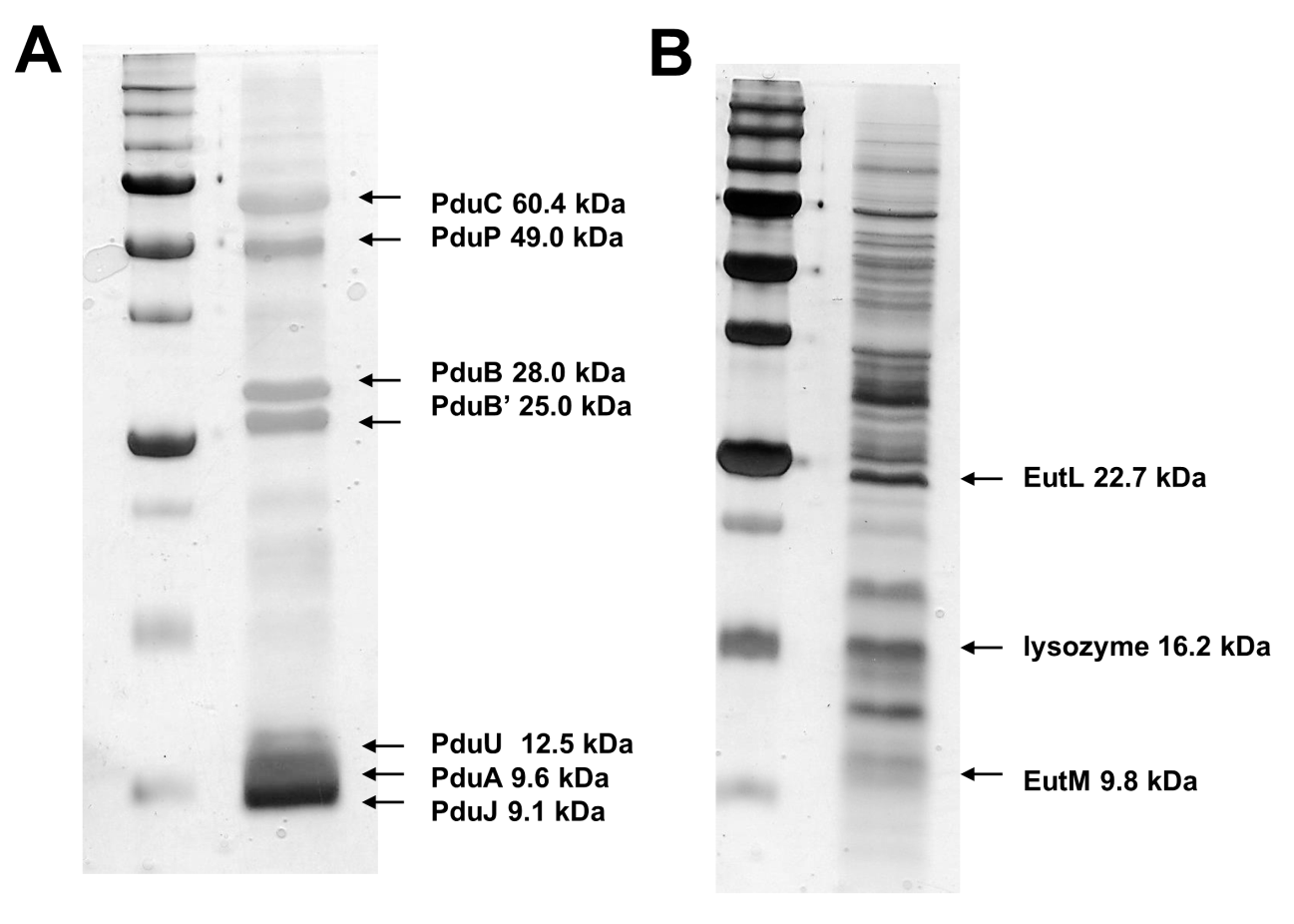


**Supplementary Figure 10. SDS-PAGE analysis of isolated native Pdu and Eut BMCs from *S. enterica* LT2.** BMCs were isolated from *S. enterica* LT2 cultures grown in E medium supplemented with 30 mM 1,2 propanediol (**A**) or ethanolamine (**B**) to induce BMC formation. Bands labelled with arrows were excised from the gel and protein identities were confirmed by LC/MS (**Supplementary Figure 11**). Predicted molecular weights of identified proteins are also provided. Lysozyme, which was added during the cell lysis procedure, serves as an internal standard.


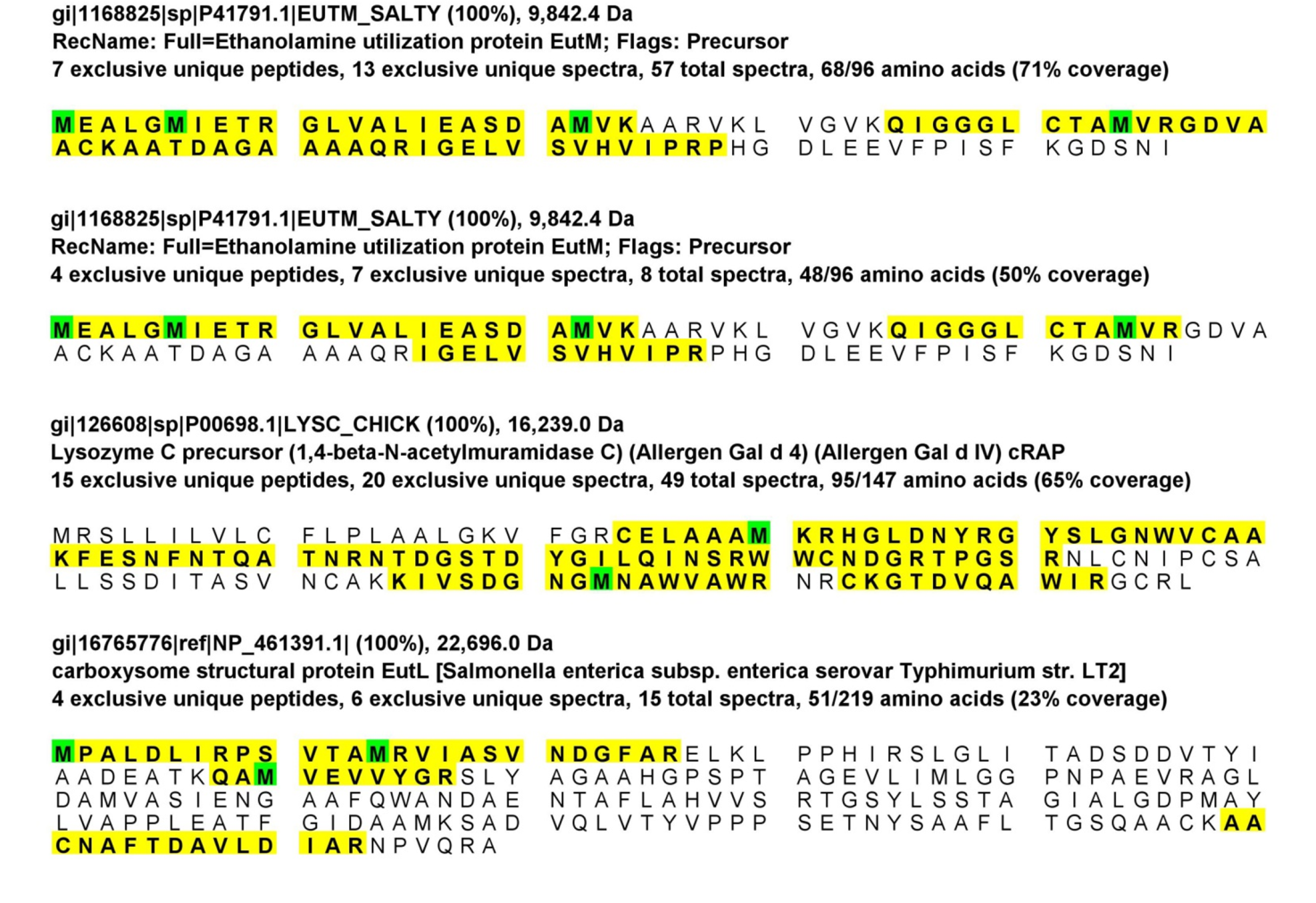


**Supplementary Figure 11. Protein identities of Eut BMC shell proteins confirmed by trypsin digest and LC/MS.** A representative example of confidence of protein identities as well as peptide coverage (highlighted in yellow) obtained from excised gel bands following SDS-PAGE analysis of isolated native Eut BMCs.


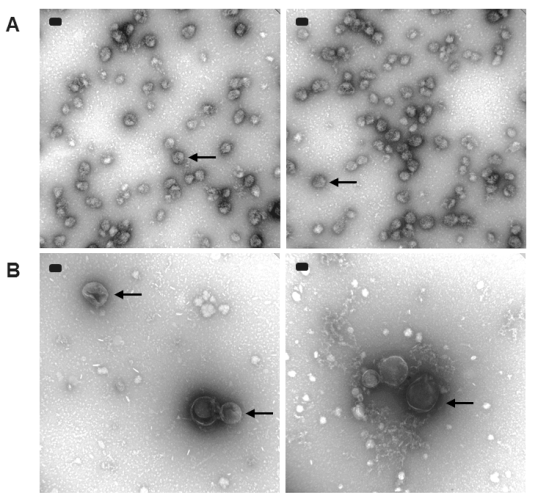


**Supplementary Figure 12. Negative stain TEM of native Pdu and Eut BMCs isolated from *S. enterica* LT2.** Pdu BMCs (**A**) and Eut BMCs (**B**) are indicated with arrows. For both samples two representative images are shown, images were taken at a magnification of x 53,000. The scale bar represents 100 nm.


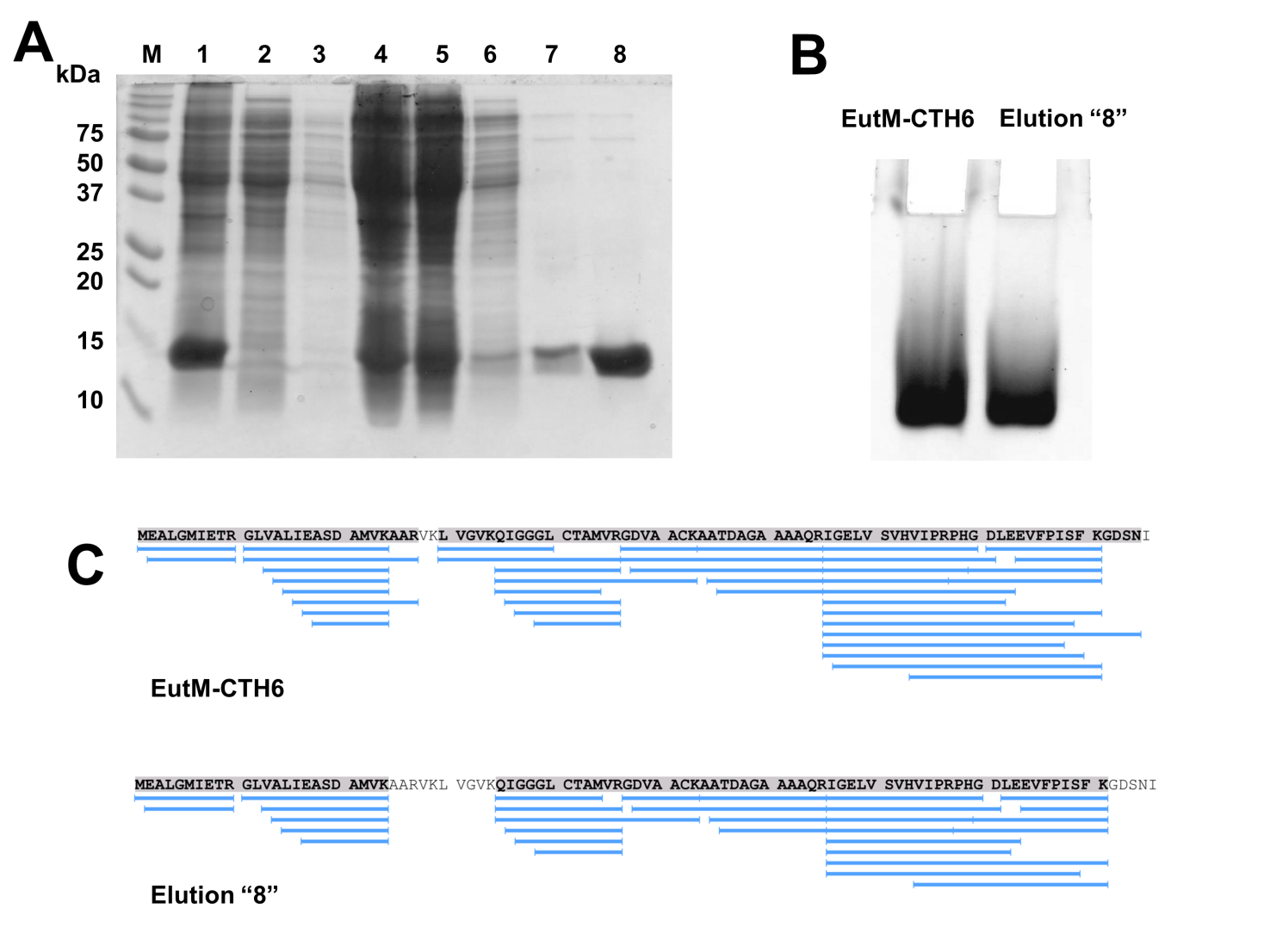


**Supplementary Figure 13. Protein pulldown between EutM-CTH6 and ∆EutQ1-100 indicates that the proteins do not interact *in vitro*.** (**A**) SDS-PAGE analysis of protein pulldowns. The lanes are labelled as follows: 1 = EutM-CTH6 soluble protein prior to loading on the Talon resin, 2 = flow through upon loading EutM-CTH6 onto the Talon resin in 5 mM imidazole buffer, 3 = removal of unbound proteins during a wash of the EutM-CTH6 loaded Talon resin with 5 mM imidazole buffer, 4 = ∆EutQ1-100 soluble protein prior to loading on the EutM-CTH6 loaded Talon resin, 5 = flow through upon loading ∆EutQ1-100 onto the EutM-CTH6 loaded Talon resin in 5 mM imidazole buffer, 6 = removal of unbound proteins during a wash of the ∆EutQ1-100 + EutM-CTH6 loaded Talon resin in 5 mM imidazole buffer, 7 = elution of protein from the Talon resin in 50 mM imidazole buffer, 8 = elution of protein from the Talon resin in 250 mM imidazole buffer. Expected protein sizes are EutM-CTH6 = 10.7 kDa, ∆EutQ1-100 = 14.2 kDa. Note that His-tagged EutM runs slightly higher than the predicted molecular weight on a 15 % SDS-PAGE gel. The lack of an eluted protein band between 10-15 kDa in lane 2 indicates that EutM-CTH6 binds completely to the Talon resin. The presence of an eluted protein band between 10-15 kDa in lane 5 indicates that ∆EutQ1-100 passes through the column and does not interact with preloaded EutM-CTH6. (**B**) Native-PAGE analysis of EutM-CTH6 and the protein(s) eluted in 250 mM imidazole from pulldown experiments (lane 8 in (**A**)). Note, ∆EutQ1-100 has a pI 4.5 and would be expected to migrate as a separate band from EutM-CTH6 (pI 6.7) under these buffer conditions (pH 8.8). However, only one band was apparent. (**C**) Peptide mass sequencing of the two protein bands excised from the native-PAGE gel in (**B**). Both bands had highest coverage of peptides corresponding to EutM (97 % and 85 % respectively); no peptides were detected corresponding to EutQ.


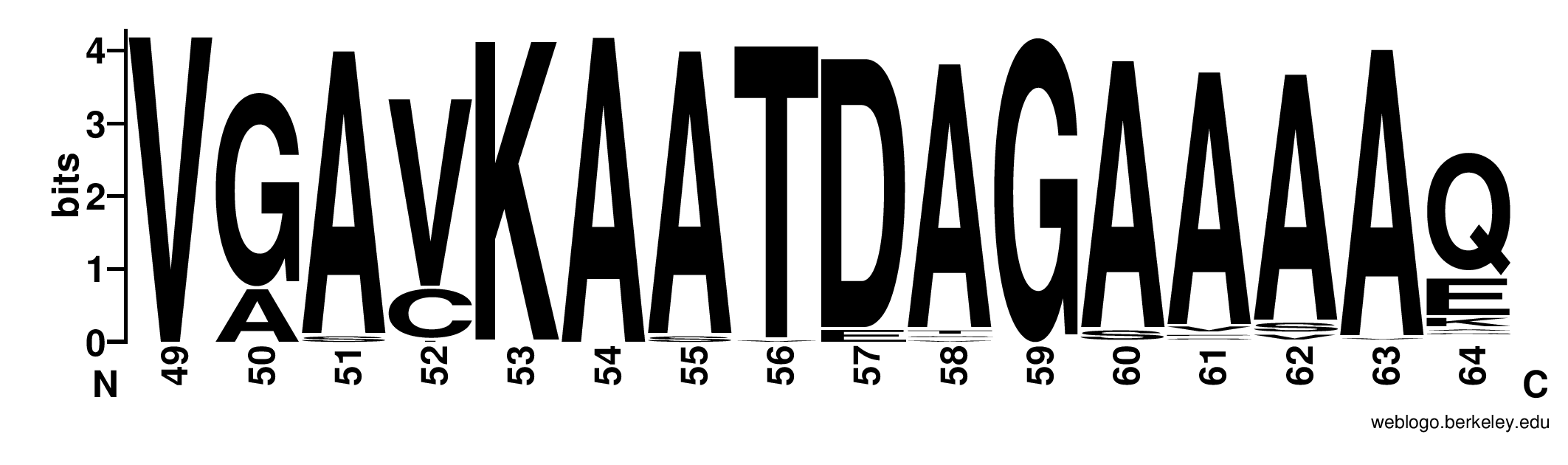


**Supplementary Figure 14. Sequence conservation of the putative EutM helix involved in EutQ interactions.** A graphical representation of sequence conservation was generated using the WebLogo server (<http://weblogo.berkeley.edu/>) based on a multiple sequence alignment generated with 138 EutM homologs as described in the Supplementary Methods. The overall height of the amino acid letter (bits) indicates the sequence conservation at that position. For clarity, only amino acids V49 – Q64 (EutM *S. enterica* LT2 numbering) are shown.

***Link to Movie 1***

**Supplementary Movie 1. *E. coli* cells coexpressing EutQ, along with EutSMNLK shell proteins produce unconstrained structures *in vivo*.** *E. coli* C2566 cells expressing EutQSMNLK form nanocompartment-like fluorescent puncta (representative puncta highlighted with an arrow) that vibrate in place. Images were collected for a total of 3min at a 3sec interval (movie shows two frames per second). Nanocompartments are tagged with the EutC^1-19^-EGFP fusion.

***Link to Movie 2***

**Supplementary Movie 2. *E. coli* cells expressing EutSMNLK shell proteins alone form a single, immobile structure *in vivo*.** *E. coli* C2566 cells expressing EutSMNLK form a single immobile nanocompartment-like fluorescent punctum per cell (representative puncta highlighted with an arrow). Images were collected for a total of 3min at a 3sec interval (movie shows two frames per second). Nanocompartments are tagged with the EutC^1-19^-EGFP fusion.

***Link to Movie 3***

**Supplementary Movie 3. Native Eut BMCs of *S. enterica* LT2.** Time-lapse imaging of native Eut BMCs formed in wild-type *S. enterica* LT2 cells grown under ethanolamine conditions reveals numerous, unconstrained fluorescent puncta *in vivo* (representative puncta highlighted with an arrow). Images were collected for a total of 3min at a 3sec interval (movie shows two frames per second). BMCs are tagged with the EutC^1-19^-EGFP fusion.

***Link to Movie 4***

**Supplementary Movie 4. *ΔEutQ*** **mutants of *S. enterica* LT2 form a single, immobile puncta.** Time-lapse imaging of native Eut BMCs formed in ∆EutQ *S. enterica* LT2 cells grown under ethanolamine conditions reveals a single immobile fluorescent punctum at the pole of cells (representative puncta highlighted with an arrow). Images were collected for a total of 3min at a 3sec interval (movie shows two frames per second). BMCs are tagged with the EutC^1-19^-EGFP fusion.

**SUPPLEMENTARY REFERENCES**

1 Vick, J. E. *et al.* Optimized compatible set of BioBrick vectors for metabolic pathway engineering. *Appl Microbiol Biotechnol* **92**, 1275-1286, (2011).

2 Brinsmade, S. R., Paldon, T. & Escalante-Semerena, J. C. Minimal functions and physiological conditions required for growth of salmonella enterica on ethanolamine in the absence of the metabolosome. *J Bacteriol* **187**, 8039-8046, (2005).

3 Altschul, S. F., Gish, W., Miller, W., Myers, E. W. & Lipman, D. J. Basic local alignment search tool. *J Mol Biol* **215**, 403-410, (1990).

4 Tsoy, O., Ravcheev, D. & Mushegian, A. Comparative genomics of ethanolamine utilization. *J. Bacteriol.* **191**, 7157-7164, (2009).

5 Larkin, M. A. *et al.* Clustal W and Clustal X version 2.0. *Bioinformatics* **23**, 2947-2948, (2007).

6 Tamura, K., Stecher, G., Peterson, D., Filipski, A. & Kumar, S. MEGA6: Molecular Evolutionary Genetics Analysis version 6.0. *Mol Biol Evol* **30**, 2725-2729, (2013).

7 Crooks, G. E., Hon, G., Chandonia, J. M. & Brenner, S. E. WebLogo: a sequence logo generator. *Genome Res* **14**, 1188-1190, (2004).

8 Takenoya, M., Nikolakakis, K. & Sagermann, M. Crystallographic insights into the pore structures and mechanisms of the EutL and EutM shell proteins of the ethanolamine-utilizing microcompartment of *Escherichia coli*. *J. Bacteriol.* **192**, 6056-6063, (2010).

9 Pitts, A. C., Tuck, L. R., Faulds-Pain, A., Lewis, R. J. & Marles-Wright, J. Structural insight into the *Clostridium difficile* ethanolamine utilization microcompartment. *PLoS One* **7**, e48360, (2012).

10 Penrod, J. T. & Roth, J. R. Conserving a volatile metabolite: a role for carboxysome-like organelles in *Salmonella enterica*. *J. Bacteriol.* **188**, 2865-2874, (2006).

11 Choudhary, S., Quin, M. B., Sanders, M. A., Johnson, E. T. & Schmidt-Dannert, C. Engineered protein nano-compartments for targeted enzyme localization. *PLoS One* **7**, e33342, (2012).
